# Fast covariance estimation for multivariate sparse functional data

**DOI:** 10.1002/sta4.245

**Published:** 2020-06-17

**Authors:** Cai Li, Luo Xiao, Sheng Luo

**Affiliations:** ^1^ Department of Statistics North Carolina State University Raleigh 27607 NC; ^2^ Department of Biostatistics and Bioinformatics Duke University Durham 27705 NC

**Keywords:** bivariate smoothing, covariance function, functional principal component analysis, longitudinal data, multivariate functional data, prediction

## Abstract

Covariance estimation is essential yet underdeveloped for analysing multivariate functional data. We propose a fast covariance estimation method for multivariate sparse functional data using bivariate penalized splines. The tensor‐product B‐spline formulation of the proposed method enables a simple spectral decomposition of the associated covariance operator and explicit expressions of the resulting eigenfunctions as linear combinations of B‐spline bases, thereby dramatically facilitating subsequent principal component analysis. We derive a fast algorithm for selecting the smoothing parameters in covariance smoothing using leave‐one‐subject‐out cross‐validation. The method is evaluated with extensive numerical studies and applied to an Alzheimer's disease study with multiple longitudinal outcomes.

## 1 INTRODUCTION

Functional data analysis (FDA) has been enjoying great successes in many applied fields, for example, neuroimaging (Reiss & Ogden, 2010; Lindquist, 2012; Goldsmith, Crainiceanu, Caffo, & Reich, 2012; Zhu, Li, & Kong, 2012), genetics (Leng & Müller, 2006; Reimherr & Nicolae, 2014; 2016), and wearable computing (Morris et al., 2006; Xiao et al., 2015). Functional principal component analysis (FPCA) conducts dimension reduction on the inherently infinite‐dimensional functional data and thus facilitates subsequent modelling and analysis. Traditionally, functional data are densely observed on a common grid and can be easily connected to multivariate data, although the notion of smoothness distinguishes the former from the latter. In recent years, covariance‐based FPCA (Yao, Müller, & Wang, 2005) has become a standard approach and has greatly expanded the applicability of functional data methods to irregularly spaced data such as longitudinal data. Various nonparametric methods have now been proposed to estimate the smooth covariance function, for example, Peng and Paul (2009), Cai and Yuan (2010), Goldsmith et al. (2012), Xiao, Li, Checkley, and Crainiceanu (2018), and Wong and Zhang (2019).

There has been growing interest in multivariate functional data where multiple functions are observed for each subject. For dense functional data, Ramsay and Silverman (2005, Chapter 8.5) proposed to concatenate multivariate functional data as a single vector and conduct multivariate PCA on the long vectors, and Berrendero, Justel, and Svarc (2011) repeatedly applied point‐wise univariate PCA. For sparse and paired functional data, Zhou, Huang, and Carroll (2008) extended the low‐rank mixed effects model in James, Hastie, and Sugar (2000). Chiou, Chen, and Yang (2014) considered normalized multivariate FPCA through standardizing the covariance operator. Petersen and Müller (2016) proposed various metrics for studying cross‐covariance between multivariate functional data. More recently, Happ and Greven (2018) introduced a FPCA framework for multivariate functional data defined on different domains.

The interest of the paper is FPCA for multivariate sparse functional data, where multiple responses are observed at time points that vary from subject to subject and may even vary between responses within subjects. There are much fewer works to handle such data. The approach in Zhou et al. (2008) focuses on bivariate functional data and can be extended to more than two‐dimensional functional data, although model selection (e.g., selection of smoothing parameters) can be computationally difficult and convergence of the expectation–maximization estimation algorithm could also be an issue. The local polynomial method in Chiou et al. (2014) can be applied to multivariate sparse functional data, although a major drawback is the selection of multiple bandwidths. Moreover, because the local polynomial method is a local approach, there is no guarantee that the resulting estimates of covariance functions will lead to a properly defined covariance operator. The approach in Happ and Greven (2018) (denoted by mFPCA hereafter) estimates cross‐covariances via scores from univariate FPCA and hence can be applied to multivariate sparse functional data. Although mFPCA is theoretically sound for dense functional data, it may not capture cross‐correlations between functions because scores from univariate FPCA for sparse functional data are shrunk towards zero.

We propose a novel and fast covariance‐based FPCA method for multivariate sparse functional data. Note that multiple auto‐covariance functions for within‐function correlations and cross‐covariance functions for between‐function correlations have to be estimated. Tensor‐product B‐splines are employed to approximate the covariance functions, and a smoothness penalty as in bivariate penalized splines (Eilers & Marx, 2003) is adopted to avoid overfit. Then, the individual estimates of covariance functions will be pooled and refined. The advantages of the new method are multifold. First, the tensor‐product B‐spline formulation is computationally efficient to handle multivariate sparse functional data. Second, a fast fitting algorithm for selecting the smoothing parameters will be derived, which alleviates the computational burden of conducting leave‐one‐subject‐out cross‐validation. Third, the tensor‐product B‐spline representation of the covariance functions enables a straightforward spectral decomposition of the covariance operator for the multivariate functional data; see Proposition 1. In particular, the eigenfunctions associated with the covariance operator are explicit functions of the B‐spline bases. Last but not the least, via a simple truncation step, the refined estimates of the covariance functions lead to a properly defined covariance operator.

Compared with mFPCA, the proposed method does not rely on scores from univariate FPCA, which could be a severe problem for sparse functional data and hence could better capture the correlations between functions. And an improved correlation estimation will lead to improved subsequent FPCA analysis and curve prediction. The proposed method also compares favourably with the local polynomial method in Chiou et al. (2014) because of the computationally efficient tensor‐product spline formulation of the covariance functions and the derived fast algorithm for selecting the smoothing parameters. Moreover, as mentioned above, there exists an explicit and easy‐to‐calculate relationship between the tensor‐product spline representation of covariance functions and the associated eigenfunctions/eigenvalues, which greatly facilitate subsequent FPCA analysis.

In addition to FPCA, there are also abundant literatures on models for multivariate functional data with most focusing on dense functional data. For clustering of multivariate functional data, see Zhu, Brown, and Morris (2012), Jacques and Preda (2014), Huang, Li, and Guan (2014), and Park and Ahn (2017). For regression with multivariate functional responses, see Zhu et al. (2012), Luo and Qi (2017), Li, Huang, and Zhu, (2017), Wong, Li, and Zhu (2019), Zhu, Morris, Wei, and Cox (2017), Kowal, Matteson, and Ruppert (2017), and Qi and Luo (2018). Graphical models for multivariate functional data are studied in (Zhu, Strawn, & Dunson, 2016) and Qiao, Guo, and James (2019). Works on multivariate functional data include also Chiou and Müller (2014, 2016).

The remainder of the paper proceeds as follows. In Section 2, we present our proposed method. We conduct extensive simulation studies in Section 3 and apply the proposed method to an Alzheimer's disease (AD) study in Section 4. A discussion is given in Section 5. All technical details are enclosed in the Appendix.

## 2 METHODS

### 2.1 Fundamentals of multivariate functional principal component analysis

Let *p* be a positive integer and denote by T a continuous and bounded domain in the real line R. Consider the Hilbert space $H:\underset{p}{\underbrace{L^{2}(T)\times \text{…}\times L^{2}(T)}}$ equipped with the inner product $<\cdot ,\cdot {>}_{H}$ and norm $\|\cdot {\|}_{H}$ such that for arbitrary functions $f={\left(f^{\left(1\right)},\text{…},f^{\left(p\right)}\right)}^{⊤}$ and $g={\left(g^{\left(1\right)},\text{…},g^{\left(p\right)}\right)}^{⊤}$ in H with each element in $L^{2}(T)$, $<f,g{>}_{H}=\sum_{k=1}^{p}\int f^{\left(k\right)}(t)g^{\left(k\right)}(t)\;dt$ and $\|f{\|}_{H}=<f,f{>}_{H}^{1/2}$. Let ${\left\{x^{\left(k\right)}\right\}}_{k=1,\text{…},p}$ be a set of *p* random functions with each function in $L^{2}(T)$. Assume that the *p*‐dimensional vector $x(t)={\left(x^{\left(1\right)},\text{…},x^{\left(p\right)}\right)}^{⊤}\in R^{p}$ has a *p*‐dimensional smooth mean function, $\mu (t)=E\{x(t)\}={\left(E\left\{x^{\left(1\right)}(t)\right\},\text{…},E\left\{x^{\left(p\right)}(t)\right\}\right)}^{⊤}={\left(\mu^{\left(1\right)}(t),\text{…},\mu^{\left(p\right)}(t)\right)}^{⊤}$. Define the covariance function as $C(s,t)=E\left\{(x(s)-\mu (s)){\left(x(t)-\mu (t)\right)}^{⊤}\right\}={\left[C_{kk^{\prime }}(s,t)\right]}_{1\leq k,k^{\prime }\leq p}$ and $C_{kk^{'}}(s,t)=\text{Cov}\left\{x^{\left(k\right)}(s),x^{\left(k^{'}\right)}(t)\right\}$. Then, the covariance operator Γ:H→H associated with the kernel **C**(*s*,*t*) can be defined such that for any f∈H, the *k*th element of **Γf** is given by

$${\left(\Gamma f\right)}^{\left(k\right)}(s)=<C_{k}(s,\cdot ),f{>}_{H}=\overset{p}{\underset{k^{\prime }=1}{\sum }}\int C_{kk^{\prime }}(s,t)f^{\left(k^{\prime }\right)}(t)\;dt,$$

where **C**_*k*_(*s*,*t*)=(*C*_*k*1_(*s*,*t*),…,*C*_*kp*_(*s*,*t*))^⊤^. Note that **Γ** is a linear, self‐adjoint, compact, and non‐negative integral operator. By the Hilbert–Schmidt theorem, there exists a set of orthonormal bases ${\left\{\Psi_{\ell }\right\}}_{\ell \geq 1}\in H$, $\Psi_{\ell }={\left(\Psi_{\ell }^{\left(1\right)},\text{…},\Psi_{\ell }^{\left(p\right)}\right)}^{⊤}$, and $<\Psi_{\ell },\Psi_{\ell^{\prime }}{>}_{H}=\sum_{k=1}^{p}\int \Psi_{\ell }^{\left(k\right)}(t)\Psi_{\ell^{\prime }}^{\left(k\right)}(t)\;dt=1_{\left\{\ell =\ell^{\prime }\right\}}$, such that

(1) $${\left(\Gamma \Psi_{\ell }\right)}^{\left(k\right)}(s)=\overset{p}{\underset{k^{\prime }=1}{\sum }}\int C_{kk^{\prime }}(s,t)\Psi_{\ell }^{\left(k^{\prime }\right)}(t)\;dt=d_{\ell }\Psi_{\ell }^{\left(k\right)}(s),$$

where *d*_*ℓ*_ is the *ℓ*th largest eigenvalue corresponding to **Ψ**_*ℓ*_. Then, the multivariate Mercer's theorem gives

(2) $$C(s,t)=\overset{\infty }{\underset{\ell }{\sum }}d_{\ell }\Psi_{\ell }(s)\Psi_{\ell }^{⊤}(t),$$

where $C_{kk^{\prime }}(s,t)=\sum_{\ell =1}^{\infty }d_{\ell }\Psi_{\ell }^{\left(k\right)}(s)\Psi_{\ell }^{\left(k^{\prime }\right)}(t)$. As shown in Saporta (1981), **x**(*t*) has the multivariate Karhunen–Loève representation, $x(t)=\mu (t)+\sum_{\ell =1}^{\infty }\xi_{\ell }\Psi_{\ell }(t)$, where $\xi_{\ell }=<x-\mu ,\Psi_{\ell }{>}_{H}$ are the scores with $E(\xi_{\ell })=0$ and $E(\xi_{\ell }\xi_{\ell^{\prime }})=d_{\ell }1_{\left\{\ell =\ell^{\prime }\right\}}$. The covariance operator **Γ** has the positive semidefiniteness property; that is, for any $a={\left(a_{1},\text{…},a_{p}\right)}^{⊤}\in R^{p}$, the covariance function of ***a*** ^⊤^ **x**, denoted by *C*_***a***_(*s*,*t*), satisfies that for any sets of time points $(t_{1},\text{…},t_{q})\subset T$ with an arbitrary positive integer *q*, the square matrix ${\left[C_{a}(t_{i},t_{j})\right]}_{\left\{1\leq i,j\leq q\right\}}\in R^{q\times q}$ is positive semidefinite.

### 2.2 Covariance estimation by bivariate penalized splines

Suppose that the observed data take the form $\left\{\left(y_{ij}^{\left(k\right)},t_{ij}^{\left(k\right)}\right):i=1,\text{…},n;\;\;k=1,\text{…},p;\;\;j=1,\text{…},m_{ik}\right\}$, where $t_{ij}^{\left(k\right)}\in T$ is the observed time point, $y_{ij}^{\left(k\right)}$ is the observed *k*th response, *n* is the number of subjects, and *m*_*ik*_ is the number of observations for subject *i*'s *k*th response. The model is

(3) $$y_{ij}^{\left(k\right)}=x_{i}^{\left(k\right)}\left(t_{ij}^{\left(k\right)}\right)+\epsilon_{ij}^{\left(k\right)}=\mu^{\left(k\right)}\left(t_{ij}^{\left(k\right)}\right)+\overset{\infty }{\underset{\ell =1}{\sum }}\xi_{i\ell }\Psi_{\ell }^{\left(k\right)}\left(t_{ij}^{\left(k\right)}\right)+\epsilon_{ij}^{\left(k\right)},$$

where $x_{i}(t)={\left(x_{i}^{\left(1\right)}(t),\text{…},x_{i}^{\left(p\right)}(t)\right)}^{⊤}\in H$, $\epsilon_{ij}^{\left(k\right)}$ are random noises with zero means and variances $\sigma_{k}^{2}$ and are independent across *i*,*j*, and *k*.

The goal is to estimate the covariance functions *C*_*kk* *′*_. We adopt a three‐step procedure. In the first step, empirical estimates of the covariance functions are constructed. Let $r_{ij}^{\left(k\right)}=y_{ij}^{\left(k\right)}-\mu^{\left(k\right)}\left(t_{ij}^{\left(k\right)}\right)$ be the residuals and $C_{ij_{1}j_{2}}^{\left(kk^{\prime }\right)}=r_{ij_{1}}^{\left(k\right)}r_{ij_{2}}^{\left(k^{\prime }\right)}$ be the auxiliary variables. Note that $E\left(C_{ij_{1}j_{2}}^{\left(kk^{\prime }\right)}\right)=C_{kk^{\prime }}\left(t_{ij_{1}}^{\left(k\right)},t_{ij_{2}}^{\left(k^{\prime }\right)}\right)+\sigma_{k}^{2}1_{\left\{k=k^{\prime },j_{1}=j_{2}\right\}}$ for 1 ≤ *j*_1_ ≤ *m*_*ik*_,1 ≤ *j*_2_ ≤ *m*_*ik* *′*_. Thus, $C_{ij_{1}j_{2}}^{\left(kk\prime \right)}$ is an unbiased estimate of $C_{kk^{\prime }}\left(t_{ij_{1}}^{\left(k\right)},t_{ij_{2}}^{\left(k^{\prime }\right)}\right)$ whenever *k*≠*k* *′* or *j*_1_≠*j*_2_. In the second step, the noisy auxiliary variables are smoothed to obtain smooth estimates of the covariance functions. For smoothing, we use bivariate *P*‐splines (Eilers & Marx, 2003) because it is an automatic smoother and is computationally simple. In the final step, we pool all estimates of the individual covariance functions and use an extra step of eigendecomposition to obtain refined estimates of covariance functions. The refined estimates lead to a covariance operator that is properly defined, that is, positive semidefinite. In practice, the mean functions *μ* ^(*k*)^s are unknown, and we estimate them using *P*‐splines (Eilers & Marx, 1996) with the smoothing parameters selected by leave‐one‐subject‐out cross‐validation; see the Supporting Information for details. Denote the estimates by ${\hat{\mu }}^{\left(k\right)}$. Let ${\hat{r}}_{ij}^{\left(k\right)}=y_{ij}^{\left(k\right)}-{\hat{\mu }}^{\left(k\right)}\left(t_{ij}^{\left(k\right)}\right)$ and ${Ĉ}_{ij_{1}j_{2}}^{\left(kk^{\prime }\right)}={\hat{r}}_{ij_{1}}^{\left(k\right)}{\hat{r}}_{ij_{2}}^{\left(k^{\prime }\right)}$, the actual auxiliary variables.

The bivariate *P*‐splines model $C_{kk^{\prime }}(s,t)$ uses tensor‐product splines $G_{kk^{\prime }}(s,t)$ for 1 ≤ *k*,*k* *′* ≤ *p*. Specifically, $G_{kk^{\prime }}(s,t)=\sum_{1\leq \gamma_{1},\gamma_{2}\leq c}\theta_{\gamma_{1}\gamma_{2}}^{\left(kk^{\prime }\right)}B_{\gamma_{1}}(s)B_{\gamma_{2}}(t)$, where $\Theta_{kk^{\prime }}={\left[\theta_{\gamma_{1}\gamma_{2}}^{\left(kk^{\prime }\right)}\right]}_{1\leq \gamma_{1},\gamma_{2}\leq c}\in R^{c\times c}$ is a coefficient matrix, {*B*_1_(·),…,*B*_*c*_(·)} is the collection of B‐spline basis functions in T, and *c* is the number of equally spaced interior knots plus the order (degree plus 1) of the B‐splines. Because $C_{kk^{\prime }}(s,t)=C_{k^{\prime }k}(t,s)=\text{Cov}\left\{x^{\left(k\right)}(s),x^{\left(k^{\prime }\right)}(t)\right\}$, it is reasonable to impose the assumption that

$$\Theta_{kk^{'}}=\Theta_{k^{'}k}^{⊤},$$

so that *G*_*kk* *′*_(*s*,*t*)=*G*_*k* *′* *k*_(*t*,*s*). Therefore, in the rest of the section, we consider only *k* ≤ *k* *′*.

Let $D\in R^{(c-2)\times c}$ denote a second‐order differencing matrix such that for a vector $a={\left(a_{1},\text{…},a_{c}\right)}^{⊤}\in R^{c}$, $Da={\left(a_{3}-2a_{2}+a_{1},a_{4}-2a_{3}+a_{2},\text{…},a_{c}-2a_{c-1}+a_{c-2}\right)}^{⊤}\in R^{c-2}$. Also let ‖·‖_*F*_ be the Frobenius norm. For the cross‐covariance function $C_{kk^{\prime }}(s,t)$ with *k*<*k* *′*, the bivariate *P*‐splines estimate the coefficient matrix **Θ**_*kk* *′*_ by ${\hat{\Theta }}_{kk^{\prime }}$, which minimizes the penalized least squares

(4) $$\overset{n}{\underset{i=1}{\sum }}\underset{1\leq j_{1}\leq m_{ik}}{\sum }\underset{1\leq j_{2}\leq m_{ik^{\prime }}}{\sum }{\left\{G_{kk^{\prime }}\left(t_{ij_{1}}^{\left(k\right)},t_{ij_{2}}^{\left(k^{\prime }\right)}\right)-{Ĉ}_{ij_{1}j_{2}}^{\left(kk^{\prime }\right)}\right\}}^{2}+\lambda_{kk^{\prime }1}\|D\Theta_{kk^{\prime }}{\|}_{F}^{2}+\lambda_{kk^{\prime }2}\|D\Theta_{kk^{\prime }}^{⊤}{\|}_{F}^{2},$$

where $\lambda_{kk^{\prime }1}$ and $\lambda_{kk^{\prime }2}$ are two non‐negative smoothing parameters that balance the model fit and smoothness of the estimate and will be determined later. Indeed, the column penalty $\|D\Theta_{kk^{\prime }}{\|}_{F}^{2}$ penalizes the second‐order consecutive differences of the columns of **Θ**_*kk* *′*_ and similarly, the row penalty $\|D\Theta_{kk^{\prime }}^{⊤}{\|}_{F}^{2}$ penalizes the second‐order consecutive differences of the rows of **Θ**_*kk* *′*_. The two penalty terms are essentially penalizing the second‐order partial derivatives of *G*_*kk* *′*_(*s*,*t*) along the *s* and *t* directions. The two smoothing parameters are allowed to differ to accommodate different levels of smoothing along the two directions.

For the auto‐covariance functions *C*_*kk*_(*s*,*t*) with *k*=1,…,*p*, we conduct bivariate covariance smoothing by enforcing the following constraint on the coefficient matrix **Θ**_*kk*_ (Xiao et al., 2018):

(5) $$\Theta_{kk}=\Theta_{kk}^{⊤}.$$

It follows that *G*_*kk*_(*s*,*t*) is a symmetric function. Then, the coefficient matrix **Θ**_*kk*_ and the error variance $\sigma_{k}^{2}$ are jointly estimated by ${\hat{\Theta }}_{kk}$ and ${\hat{\sigma }}_{k}^{2}$, which minimize the penalized least squares

(6) $$\overset{n}{\underset{i=1}{\sum }}\underset{1\leq j_{1},j_{2}\leq m_{ik}}{\sum }{\left\{G_{kk}\left(t_{ij_{1}}^{\left(k\right)},t_{ij_{2}}^{\left(k\right)}\right)+\sigma_{k}^{2}1_{\left\{j_{1}=j_{2}\right\}}-{Ĉ}_{ij_{1}j_{2}}^{\left(kk\right)}\right\}}^{2}+\lambda_{k}\|D\Theta_{kk}{\|}_{F}^{2},$$

over all symmetric **Θ**_*kk*_ and *λ*_*k*_ is a smoothing parameter. Note that the two penalty terms in Equation (4) become the same when **Θ**_*kk*_ is symmetric, and thus, only one smoothing parameter is needed for auto‐covariance estimation.

#### 2.2.1 Estimation

We first introduce the notation. Let vec(·) be an operator that stacks the columns of a matrix into a column vector and denote by ⊗ the Kronecker product. Fix *k* and *k* *′* with *k* ≤ *k* *′*. Let $\theta_{kk^{\prime }}=\text{vec}(\Theta_{kk^{\prime }})\in R^{c^{2}}$ be a vector of the coefficients and $b(t)={\left\{B_{1}(t),\text{…},B_{c}(t)\right\}}^{⊤}\in R^{c}$ denotes the B‐spline base. Then,

$$G_{kk^{\prime }}(s,t)=b{\left(s\right)}^{⊤}\Theta_{kk^{\prime }}b(t)={\left\{b(t)⊗b(s)\right\}}^{⊤}\theta_{kk^{\prime }}.$$

We now organize the auxiliary responses ${Ĉ}_{ij_{1}j_{2}}^{\left(kk\prime \right)}$ for each pair of *k* and *k* *′*. Let $r_{i}^{\left(k\right)}={\left(r_{i1}^{\left(k\right)},\text{…},r_{im_{ik}}^{\left(k\right)}\right)}^{⊤}\in R^{m_{ik}}$, ${\hat{C}}_{i}^{\left(kk^{\prime }\right)}=r_{i}^{\left(k\right)}⊗r_{i}^{\left(k^{\prime }\right)}\in R^{m_{ik}m_{ik^{\prime }}}$ and ${\hat{C}}^{\left(kk^{\prime }\right)}={\left({\hat{C}}_{1}^{(kk^{\prime }),⊤},\text{…},{\hat{C}}_{n}^{(kk^{\prime }),⊤}\right)}^{⊤}\in R^{N_{kk^{\prime }}}$, where $N_{kk^{\prime }}=\sum_{i=1}^{n}m_{ik}m_{ik^{\prime }}$ is the total number of auxiliary responses for the pair of *k* and *k* *′*. As for the B‐splines, let $b_{i}^{\left(k\right)}=\left[b\left(t_{i1}^{\left(k\right)}\right),\text{…},b\left(t_{im_{ik}}^{\left(k\right)}\right)\right]\in R^{c\times m_{ik}}$, $B_{i}^{\left(kk\prime \right)}={\left(b_{i}^{\left(k\prime \right)}⊗b_{i}^{\left(k\right)}\right)}^{⊤}\in R^{(m_{ik}m_{ik\prime })\times c^{2}}$, and $B^{\left(kk\prime \right)}={\left[B_{1}^{(kk\prime ),⊤},\text{…},B_{n}^{(kk\prime ),⊤}\right]}^{⊤}\in R^{N_{kk\prime }\times c^{2}}$.

For estimation of the cross‐covariance functions *C*_*kk* *′*_ with *k*<*k* *′*, the penalized least squares in Equation (4) can be rewritten as

(7) $${\left({\hat{C}}^{\left(kk^{\prime }\right)}-B^{\left(kk^{\prime }\right)}\theta_{kk^{\prime }}\right)}^{⊤}\left({\hat{C}}^{\left(kk^{\prime }\right)}-B^{\left(kk^{\prime }\right)}\theta_{kk^{\prime }}\right)+\lambda_{kk^{\prime }1}\theta_{kk^{\prime }}^{⊤}P_{1}\theta_{kk^{\prime }}+\lambda_{kk^{\prime }2}\theta_{kk^{\prime }}^{⊤}P_{2}\theta_{kk^{\prime }},$$

where $P_{1}=I_{c}⊗D^{⊤}D$ and $P_{2}=D^{⊤}D⊗I_{c}$. The expression in Equation (7) is a quadratic function of the coefficient vector ***θ***_*kk* *′*_. Therefore, we derive that

$${\hat{\theta }}_{kk^{\prime }}={\left(B^{(kk^{\prime }),⊤}B^{\left(kk^{\prime }\right)}+\lambda_{kk^{\prime }1}P_{1}+\lambda_{kk^{\prime }2}P_{2}\right)}^{-1}B^{(kk^{\prime }),⊤}{\hat{C}}^{\left(kk^{\prime }\right)}$$

and the estimate of the cross‐covariance function *C*_*kk* *′*_(*s*,*t*) is ${Ĉ}_{kk^{\prime }}(s,t)={\left\{b(t)⊗b(s)\right\}}^{⊤}{\hat{\theta }}_{kk^{\prime }}$.

For estimation of the auto‐covariance functions, because of the constraint on the coefficient matrix in Equation  (5), let $\chi_{k}\in R^{c(c+1)/2}$ be a vector obtained by stacking the columns of the lower triangle of **Θ**_*kk*_, and let $G_{c}\in R^{c^{2}\times c(c+1)/2}$ be a duplication matrix such that ***θ***_*kk*_=**G**_*c*_ *η*_*k*_ (Seber, 2008, p. 246).

Let $Z_{i}^{\left(k\right)}=\text{vec}\;(I_{m_{ik}})\in R^{m_{ik}^{2}}$ and $Z^{\left(k\right)}={\left(Z_{1}^{(k),⊤},\text{…},Z_{n}^{(k),⊤}\right)}^{⊤}\in R^{N_{kk}}$. Finally, let $\beta_{k}={\left(\chi_{k}^{⊤},\sigma_{k}^{2}\right)}^{⊤}\in R^{\tilde{c}}$ with $\tilde{c}=c(c+1)/2+1$. It follows that the penalized least squares in Equation (6) can be rewritten as

$${\left({\hat{C}}^{\left(kk\right)}-X^{\left(k\right)}\beta_{k}\right)}^{⊤}\left({\hat{C}}^{\left(kk\right)}-X^{\left(k\right)}\beta_{k}\right)+\lambda_{k}\beta_{k}^{⊤}Q\beta_{k},$$

where $X^{\left(k\right)}=\left[B^{\left(kk\right)},Z^{\left(k\right)}\right]\in R^{N_{kk}\times \tilde{c}}$ and $Q=\text{blockdiag}\left\{G_{c}^{⊤}(I_{c}⊗DD^{⊤})G_{c}^{⊤},0\right\}\in R^{\tilde{c}\times \tilde{c}}$. Therefore, we obtain

$${\hat{\beta }}_{k}={\left({\hat{\chi }}_{k}^{⊤},{\hat{\sigma }}_{k}^{2}\right)}^{⊤}={\left(X^{(k),⊤}X^{\left(k\right)}+\lambda_{k}Q\right)}^{-1}X^{(k),⊤}{\hat{C}}^{\left(kk\right)}.$$

It follows that ${\hat{\theta }}_{kk}=G_{c}{\hat{\chi }}_{k}$, and the estimate of the auto‐covariance function *C*_*kk*_(*s*,*t*) is ${Ĉ}_{kk}(s,t)={\left\{b(t)⊗b(s)\right\}}^{⊤}{\hat{\theta }}_{kk}$.

The above estimates of covariance functions may not lead to a positive semidefinite covariance operator and thus have to be refined. We pool all estimates together, and we shall use the following proposition.

Proposition 1 Assume that $C_{kk^{\prime }}(s,t)=b{\left(s\right)}^{⊤}\Theta_{kk^{\prime }}b(t)$. Let $G=\int b(t)b{\left(t\right)}^{⊤}\;dt\in R^{c\times c}$ and assume that **G** is positive definite (Zhou, Shen, & Wolfe, 1998). Then, ${\left[G^{\frac{1}{2}}\Theta_{kk^{\prime }}G^{\frac{1}{2}}\right]}_{1\leq k,k^{\prime }\leq p}\in R^{pc\times pc}$ admits the spectral decomposition, $\sum_{\ell =1}^{\infty }d_{\ell }u_{\ell }u_{\ell }^{⊤}$, where *d*_*ℓ*_ is the *ℓ*th largest eigenvalue of the covariance operator **Γ**, and $u_{\ell }={\left\{u_{\ell }^{(1),⊤},\text{…},u_{\ell }^{(p),⊤}\right\}}^{⊤}\in R^{pc}$ is the associated eigenvector with $u_{\ell }^{\left(k\right)}\in R^{c}$ and such that $\Psi_{\ell }^{\left(k\right)}(t)=b{\left(t\right)}^{⊤}G^{-\frac{1}{2}}u_{\ell }^{\left(k\right)}$.

The proof is provided in Appendix A. Proposition 1 implies that, with the tensor‐product B‐spline representation of the covariance functions, one spectral decomposition gives us the eigenvalues and eigenfunctions. In particular, the eigenfunctions $\Psi_{\ell }^{\left(k\right)}(t)$ are linear combinations of the B‐spline basis functions, which means that they can be straightforwardly evaluated, an advantage of spline‐based methods compared with other smoothing methods for which eigenfunctions are approximated by spectral decompositions of the covariance functions evaluated at a grid of time points.

Once we have ${\hat{\Theta }}_{kk^{\prime }}$, the estimate of the coefficient matrix **Θ**_*kk* *′*_, the spectral decomposition of ${\left[G^{\frac{1}{2}}{\hat{\Theta }}_{kk^{\prime }}G^{\frac{1}{2}}\right]}_{kk^{\prime }}$ gives us estimates ${\hat{d}}_{\ell }$ and ${\hat{u}}_{\ell }={\left\{{\hat{u}}_{\ell }^{(1),⊤},\text{…},{\hat{u}}_{\ell }^{(p),⊤}\right\}}^{⊤}$. We discard negative ${\hat{d}}_{\ell }$ to ensure that the multivariate covariance operator is positive semidefinite, and this leads to a refined estimate of the coefficient matrix **Θ**_*kk* *′*_, ${\tilde{\Theta }}_{kk^{\prime }}=G^{-\frac{1}{2}}\left\{\sum_{\ell :{\hat{d}}_{\ell }>0}{\hat{d}}_{\ell }{\hat{u}}_{\ell }^{\left(k\right)}{\hat{u}}_{\ell }^{(k^{\prime }),⊤}\right\}G^{-\frac{1}{2}}$. Then, the refined estimate of the covariance functions is ${\tilde{C}}_{kk^{\prime }}(s,t)=b{\left(s\right)}^{⊤}{\tilde{\Theta }}_{kk^{\prime }}b(t)$. Proposition 1 also suggests that the eigenfunctions can be estimated by ${\tilde{\Psi }}_{\ell }^{\left(k\right)}(t)=b{\left(t\right)}^{⊤}G^{-\frac{1}{2}}{\hat{u}}_{\ell }^{\left(k\right)}$.

For principal component analysis or curve prediction in practice, one may select further the number of principal components by either the proportion of variance explained (PVE) (Greven, Crainiceanu, Caffo, & Reich, 2010) or an Akaike information criterion‐type criterion (Li, Wang, & Carroll, 2013). Here, we follow Greven et al. (2010) using PVE with a value of 0.99.

#### 2.2.2 Selection of smoothing parameters

We select the smoothing parameters in each auto‐covariance/cross‐covariance estimation using leave‐one‐subject‐out cross‐validation; see, for example, Yao et al. (2005) and Xiao et al. (2018). A fast approximate algorithm for the auto‐covariance has been derived in Xiao et al. (2018). So we focus on the cross‐covariance and use the notation in Equation (7). Note that there are two smoothing parameters for each cross‐covariance estimation.

For simplicity, we suppress the superscript and subscript *kk* *′* in Equation (7) for both $\hat{C}$ and **B**. Let ${\tilde{C}}_{i}^{\left[i\right]}$ be the prediction of the auxiliary responses ${\hat{C}}_{i}$ from the estimate using data without the *i*th subject. Let ‖·‖ be the Euclidean norm, and the cross‐validation error is

(8) $$\text{iCV}=\overset{n}{\underset{i=1}{\sum }}{\left\|{\hat{C}}_{i}-{\tilde{C}}_{i}^{\left[i\right]}\right\|}^{2}.$$

We shall also now suppress the subscript *k* from *m*_*ik*_ and *kk* *′* from *N*_*kk* *′*_. Let $S=B{\left(B^{⊤}B+\lambda_{1}P_{1}+\lambda_{2}P_{2}\right)}^{-1}B^{⊤}\in R^{N\times N}$, $S_{i}=B_{i}{\left(B^{⊤}B+\lambda_{1}P_{1}+\lambda_{2}P_{2}\right)}^{-1}B^{⊤}\in R^{m_{i}^{2}\times N}$, and $S_{ii}=B_{i}{\left(B^{⊤}B+\lambda_{1}P_{1}+\lambda_{2}P_{2}\right)}^{-1}B_{i}^{⊤}\in R^{m_{i}^{2}\times m_{i}^{2}}$.

Then, a shortcut formula for Equation (8) is

$$\text{iCV}=\overset{n}{\underset{i=1}{\sum }}{\left\|{\left(I_{m_{i}^{2}}-S_{ii}\right)}^{-1}(S_{i}\hat{C}-{\hat{C}}_{i})\right\|}^{2}.$$

Similar to Xu and Huang (2012) and Xiao et al. (2018), the iCV can be further simplified by adopting the approximation ${\left(I_{m_{i}^{2}}-S_{ii}\right)}^{-2}=I_{m_{i}^{2}}+2S_{ii}$, which results in the generalized cross‐validation, denoted by iGCV,

(9) $$\text{iGCV}={\left\|\hat{C}-S\hat{C}\right\|}^{2}+2\overset{n}{\underset{i=1}{\sum }}{\left(S_{i}\hat{C}-{\hat{C}}_{i}\right)}^{⊤}S_{ii}\left(S_{i}\hat{C}-{\hat{C}}_{i}\right).$$

Although iGCV is much easier to compute than iCV, the formula in Equation (9) is still computationally expensive to compute. Indeed, the smoother matrix **S** is of dimension 2,500×2,500 if *n*=100 and *m*_*i*_=*m*=5 for all *i*. Thus, we need to further simplify the formula.

Let $G_{n}=B^{⊤}B$, $\tilde{B}=BG_{n}^{-1/2}\in R^{N\times c^{2}}$, ${\tilde{B}}_{i}=B_{i}G_{n}^{-1/2}\in R^{m_{i}^{2}\times c^{2}}$, $f={\tilde{B}}^{⊤}\hat{C}\in R^{c^{2}}$, $f_{i}={\tilde{B}}_{i}^{⊤}{\hat{C}}_{i}\in R^{c^{2}}$, and $L_{i}={\tilde{B}}_{i}^{⊤}{\tilde{B}}_{i}\in R^{c^{2}\times c^{2}}$. Also let ${\tilde{P}}_{1}=G_{n}^{-1/2}P_{1}G_{n}^{-1/2}\in R^{c^{2}\times c^{2}}$, ${\tilde{P}}_{2}=G_{n}^{-1/2}P_{2}G_{n}^{-1/2}\in R^{c^{2}\times c^{2}}$, and $\sum =I_{c^{2}}+\lambda_{1}{\tilde{P}}_{1}+\lambda_{2}{\tilde{P}}_{2}$. Then, Equation (9) can be simplified as

(10) $$\text{iGCV}={\left\|\hat{C}\right\|}^{2}-2f^{⊤}\sum^{-1}f+f^{⊤}\sum^{-2}f+2\overset{n}{\underset{i=1}{\sum }}{\left(L_{i}\sum^{-1}f-f_{i}\right)}^{⊤}\sum^{-1}\left(L_{i}\sum^{-1}f-f_{i}\right).$$

Note that **Σ** has two smoothing parameters. Following Wood (2000), we use an equivalent parameterization $\sum =I+\rho \{w{\tilde{P}}_{1}+(1-w){\tilde{P}}_{2}\}$, where *ρ*=*λ*_1_+*λ*_2_ represents the overall smoothing level and *w*=*λ*_1_ *ρ* ^−1^∈[0,1] is the relative weight of *λ*_1_. We conduct a two‐dimensional grid search of (*ρ*,*w*), as follows. For a given *w*, let **U**diag(**s**)**U** ^⊤^ be the eigendecompsition of $w{\tilde{P}}_{1}+(1-w){\tilde{P}}_{2}$, where $U\in R^{c^{2}\times c^{2}}$ is an orthonormal matrix and $s=(s_{1},\text{…},s_{c^{2}})\in R^{c^{2}}$ is the vector of eigenvalues. Then, $\sum^{-1}=U\text{diag}(\tilde{d})U^{⊤}$ with $\tilde{d}=1/(1+\rho s)\in R^{c^{2}}$.

Proposition 2 Let ⊙ stand for the point‐wise multiplication. Then,

$$\text{iGCV}={\left\|\hat{C}\right\|}^{2}+{\left(\tilde{f}⊙\tilde{d}\right)}^{⊤}(\tilde{f}⊙\tilde{d})-2{\tilde{d}}^{⊤}g-4{\tilde{d}}^{⊤}F\tilde{d}+2{\tilde{d}}^{⊤}\left[\overset{n}{\underset{i=1}{\sum }}\left\{{\tilde{L}}_{i}(\tilde{f}⊙\tilde{d})\right\}⊙\left\{{\tilde{L}}_{i}(\tilde{f}⊙\tilde{d})\right\}\right],$$

where ${\tilde{f}}_{i}=U^{⊤}f_{i}\in R^{c^{2}}$, $\tilde{f}=U^{⊤}f\in R^{c^{2}}$, $g=\tilde{f}⊙\tilde{f}-\sum_{i=1}^{n}{\tilde{f}}_{i}⊙{\tilde{f}}_{i}\in R^{c^{2}}$, ${\tilde{L}}_{i}=U^{⊤}L_{i}U\in R^{c^{2}\times c^{2}}$, and $F=\sum_{i=1}^{n}({\tilde{f}}_{i}{\tilde{f}}^{⊤})⊙{\tilde{L}}_{i}\in R^{c^{2}\times c^{2}}$.

The proof is provided in Appendix A. For each *w*, note that only $\tilde{d}$ depends on *ρ* and needs to be calculated repeatedly, and all other terms need to be calculated only once. The entire algorithm is presented in Algorithm 1. We give an evaluation of the complexity of the proposed algorithm. Assume that *m*_*i*_=*m* for all *i*. The first initialization (Step 1) requires *O*(*nm* ^2^ *c* ^2^+*nc* ^4^+*c* ^6^) computations. For each *w*, the second initialization (Step 2) also requires $O\{nc^{4}\min(m^{2},c^{2})+c^{6}\}$ computations. For each *ρ*, Steps 3–8 require *O*(*nc* ^4^) computations. Therefore, the formula in Proposition 2 is most efficient to calculate for sparse data with small numbers of observations per subject; that is, *m*_*i*_s are small.

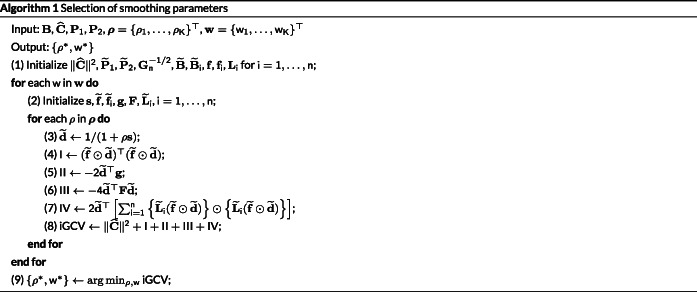

### 2.3 Prediction

For prediction, assume that the smooth curve **x**_*i*_(*t*) is generated from a multivariate Gaussian process. Suppose that we want to predict the *i*th multivariate response **x**_*i*_(*t*) at {*s*_*i*1_,…,*s*_*im*_} for *m* ≥ 1. Let $y_{i}^{\left(k\right)}={\left(y_{i1}^{\left(k\right)},\text{…},y_{im_{ik}}^{\left(k\right)}\right)}^{⊤}$ be the vector of observations at $\left\{t_{i1}^{\left(k\right)},\text{…},t_{im_{ik}}^{\left(k\right)}\right\}$ for the *k*th response. Let $\mu_{i}^{(k),o}={\left(\mu^{\left(k\right)}\left(t_{i1}^{\left(k\right)}\right),\text{…},\mu^{\left(k\right)}\left(t_{im_{ik}}^{\left(k\right)}\right)\right)}^{⊤}$ be the vector of the *k*th mean function at the observed time points. Let $y_{i}={\left(y_{i}^{(1),⊤},\text{…},y_{i}^{(p),⊤}\right)}^{⊤}$ and $\mu_{i}^{o}={\left(\mu_{i}^{(1),o,⊤},\text{…},\mu_{i}^{(p),o,⊤}\right)}^{⊤}$. Let $\mu_{i}^{n}={\left(\mu^{\left(1\right)}(s_{i1}),\text{…},\mu^{\left(1\right)}(s_{im}),\text{…},\mu^{\left(p\right)}(s_{i1}),\text{…},\mu^{\left(p\right)}(s_{im})\right)}^{⊤}$ be the vector of mean functions at the time points for prediction.

It follows that

$$\left(\begin{array}{c} y_{i}\\ x_{i} \end{array}\right)∼N\left\{\left(\begin{array}{c} \mu_{i}^{o}\\ \mu_{i}^{n} \end{array}\right),\left(\begin{array}{cc} \text{Cov}(y_{i}) & \text{Cov}{\left(x_{i},y_{i}\right)}^{⊤}\\ \text{Cov}(x_{i},y_{i}) & \text{Cov}(x_{i}) \end{array}\right)\right\}.$$

Thus, we obtain

$$E(x_{i}|y_{i})=\text{Cov}(x_{i},y_{i})\text{Cov}{\left(y_{i}\right)}^{-1}(y_{i}-\mu_{i}^{o})+\mu_{i}^{n},$$

$$\text{Cov}(x_{i}|y_{i})=\text{Cov}(x_{i})-\text{Cov}(x_{i},y_{i})\text{Cov}{\left(y_{i}\right)}^{-1}\text{Cov}{\left(x_{i},y_{i}\right)}^{⊤}.$$

Let $b_{i}^{(k),o}={\left[b\left(t_{i1}^{\left(k\right)}\right),\text{…},b\left(t_{im_{ik}}^{\left(k\right)}\right)\right]}^{⊤}$ and $b_{i}^{n}={\left[b\left(s_{i1}\right),\text{…},b\left(s_{im}\right)\right]}^{⊤}$. Next, let $B_{i}^{o}=\text{blockdiag}\left(b_{i}^{(1),o},\text{…},b_{i}^{(p),o}\right)$ and $B_{i}^{n}=I_{p}⊗b_{i}^{n}$. Then, Cov(**y**_*i*_) given by $B_{i}^{o}\Theta B_{i}^{o,⊤}+\text{blockdiag}(\sigma_{1}^{2}I_{m_{i1}},\text{…},\sigma_{p}^{2}I_{m_{ip}})$, and Cov(**x**_*i*_) and Cov(**y**_*i*_,**x**_*i*_) are given by $B_{i}^{n}\Theta B_{i}^{n,⊤}$ and $B_{i}^{o}\Theta B_{i}^{n,⊤}$, respectively. Let $\tilde{\Theta }={\left[{\tilde{\Theta }}_{kk^{\prime }}\right]}_{1\leq k,k^{\prime }\leq p}\in R^{pc\times pc}$. Plugging in the estimates, we predict **x**_*i*_ by

$$\begin{array}{ccc} {\hat{x}}_{i} & ={\left({\hat{x}}_{i}^{\left(1\right)}(s_{i1}),\text{…},{\hat{x}}_{i}^{\left(1\right)}(s_{im}),\text{…},{\hat{x}}_{i}^{\left(p\right)}(s_{i1}),\text{…},{\hat{x}}_{i}^{\left(p\right)}(s_{im})\right)}^{⊤} & \\ & =\left(B_{i}^{n}\tilde{\Theta }B_{i}^{o,⊤}\right){\hat{V}}_{i}^{-1}(y_{i}-{\hat{\mu }}_{i}^{o})+{\hat{\mu }}_{i}^{n}, \end{array}$$

where ${\hat{\mu }}_{i}^{o}={\left({\hat{\mu }}^{\left(1\right)}\left(t_{i1}^{\left(1\right)}\right),\text{…},{\hat{\mu }}^{\left(1\right)}\left(t_{im_{i1}}^{\left(1\right)}\right),\text{…},{\hat{\mu }}^{\left(p\right)}\left(t_{i1}^{\left(p\right)}\right),\text{…},{\hat{\mu }}^{\left(p\right)}\left(t_{im_{ip}}^{\left(p\right)}\right)\right)}^{⊤}$ is the estimate of $\mu_{i}^{o}$, ${\hat{\mu }}_{i}^{n}={\left({\hat{\mu }}^{\left(1\right)}(s_{i1}),\text{…},{\hat{\mu }}^{\left(1\right)}(s_{im}),\text{…},{\hat{\mu }}^{\left(p\right)}(s_{i1}),\text{…},{\hat{\mu }}^{\left(p\right)}(s_{im})\right)}^{⊤}$ is the estimate of $\mu_{i}^{n}$, ${\hat{V}}_{i}=B_{i}^{o}\tilde{\Theta }B_{i}^{o,⊤}+\text{blockdiag}\left({\hat{\sigma }}_{1}^{2}I_{m_{i1}},\text{…},{\hat{\sigma }}_{p}^{2}I_{m_{ip}}\right)$. An approximate covariance matrix for ${\hat{x}}_{i}$ is

$$\hat{\text{Cov}}(x_{i}|y_{i})=B_{i}^{n}\tilde{\Theta }B_{i}^{n,⊤}-\left(B_{i}^{n}\tilde{\Theta }B_{i}^{o,⊤}\right){\hat{V}}_{i}^{-1}{\left(B_{i}^{n}\tilde{\Theta }B_{i}^{o,⊤}\right)}^{⊤}.$$

Therefore, a 95*%* point‐wise confidence interval for the *k*th response is given by

$${\hat{x}}_{i}^{\left(k\right)}(s_{ij})\pm 1.96\sqrt{\hat{\mathrm{Var}}\left(x_{i}^{\left(k\right)}(s_{ij})|y_{i}\right)},$$

where $\hat{\mathrm{Var}}\left(x_{i}^{\left(k\right)}(s_{ij})|y_{i}\right)$ can be extracted from the diagonal of $\hat{\text{Cov}}(x_{i}|y_{i})$.

Finally, we predict the first *L* ≥ 1 scores ***ξ***_*i*_=(*ξ*_*i*1_,…,*ξ*_*iL*_)^⊤^ for the *i*th subject. Note that $\xi_{i\ell }=\int \Psi_{\ell }{\left(t\right)}^{⊤}\{x_{i}(t)-\mu (t)\}\;dt$. With a similar derivation as above, **x**_*i*_(*t*)−***μ*** (*t*) can be predicted by ${\left\{I_{p}⊗b(t)\right\}}^{⊤}\tilde{\Theta }B_{i}^{o,⊤}{\hat{V}}_{i}^{-1}(y_{i}-{\hat{\mu }}_{i}^{o})$. By Proposition 1, the eigenfunctions $\Psi_{\ell }^{\left(k\right)}(t)$ are estimated by $b{\left(t\right)}^{⊤}G^{-\frac{1}{2}}{\hat{u}}_{\ell }^{\left(k\right)}$, and thus, $\Psi_{\ell }{\left(t\right)}^{⊤}={\hat{u}}_{\ell }^{⊤}\{I_{p}⊗G^{-\frac{1}{2}}b(t)\}$. It follows that

$${\hat{\xi }}_{i\ell }={\hat{u}}_{\ell }^{⊤}\left(I_{p}⊗G^{\frac{1}{2}}\right)\tilde{\Theta }B_{i}^{o,⊤}{\hat{V}}_{i}^{-1}(y_{i}-{\hat{\mu }}_{i}^{o}).$$

## 3 SIMULATIONS

We evaluate the finite sample performance of the proposed method (denoted by mFACEs) against mFPCA via a synthetic simulation study and a simulation study mimicking the Alzheimer's Disease Neuroimaging Initiative (ADNI) data in the real data example. Here, we report the details and results of the former as the conclusions remain the same for the latter, and details are provided in the Supporting Information.

### 3.1 Simulation settings and evaluation criteria

We generate data by model  (3) with *p*=3 responses. The mean functions are $\mu (t)={\left[5\sin(2\pi t),5\cos(2\pi t),5{\left(t-1\right)}^{2}\right]}^{⊤}$. We first specify the auto‐covariance functions. Let $\Phi_{1}(t)={\left[\sqrt{2}\sin(2\pi t),\sqrt{2}\cos(4\pi t),\sqrt{2}\sin(4\pi t)\right]}^{⊤}$, $\Phi_{2}(t)={\left[\sqrt{2}\cos(\pi t),\sqrt{2}\cos(2\pi t),\sqrt{2}\cos(3\pi t)\right]}^{⊤}$, and $\Phi_{3}(t)={\left[\sqrt{2}\sin(\pi t),\sqrt{2}\sin(2\pi t),\sqrt{2}\sin(3\pi t)\right]}^{⊤}$. Also let

$$\Lambda_{11}=\left(\begin{array}{ccc} 3 & 0 & 0\\ 0 & 1.5 & 0\\ 0 & 0 & 0.75 \end{array}\right),\;\;\Lambda_{22}=\left(\begin{array}{ccc} 3.5 & 0 & 0\\ 0 & 1.75 & 0\\ 0 & 0 & 0.5 \end{array}\right),\;\;\Lambda_{33}=\left(\begin{array}{ccc} 2.5 & 0 & 0\\ 0 & 2 & 0\\ 0 & 0 & 1 \end{array}\right).$$

Then, the auto‐covariance functions are $C_{kk}(s,t)=\Phi_{k}{\left(s\right)}^{⊤}\Lambda_{kk}\Phi_{k}(t),k=1,2,3$. For the cross‐covariance functions, let $C_{kk^{\prime }}(s,t)=\rho \Phi_{k}{\left(s\right)}^{⊤}\Lambda_{kk}^{\frac{1}{2}}\Lambda_{k^{\prime }k^{\prime }}^{\frac{1}{2}}\Phi_{k^{\prime }}(t)$ for *k*≠*k* *′*, where *ρ*∈[0,1] is a parameter to be specified. The induced covariance operator from the above specifications is proper; see Lemma 1 in Appendix B. It is easy to derive that the absolute value of cross‐correlation $\rho_{kk^{\prime }}(s,t)=C_{kk^{\prime }}(s,t)/\sqrt{C_{kk}(s,s)C_{k^{\prime }k^{\prime }}(t,t)}$ is bounded by *ρ*. Hence, *ρ* controls the overall level of correlation between responses: if *ρ*=0, then the responses are uncorrelated from each other.

The eigendecomposition of the multivariate covariance function gives nine nonzero eigenvalues with associated multivariate eigenfunctions; hence, for *ℓ*=1,…,9, we simulate the scores *ξ*_*iℓ*_ from $N(0,d_{\ell })$, where *d*_*ℓ*_ are the induced eigenvalues. Next, we simulate the white noises $\epsilon_{ij}^{\left(k\right)}$ from $N(0,\sigma_{\epsilon }^{2})$, where $\sigma_{\epsilon }^{2}$ is determined according to the signal‐to‐noise ratio $\text{SNR}=\sum_{\ell }d_{\ell }/(p\sigma_{\epsilon }^{2})$. Here, we let SNR=2. For each response, the sampling time points are drawn from a uniform distribution in the unit interval, and the number of observations for each subject, *m*_*ik*_, is generated from a uniform discrete distribution on {3,4,5,6,7}. Thus, the sampling points vary not only from subject to subject but also across responses within each subject.

We use a factorial design with two factors: the number of subjects *n* and the correlation parameter *ρ*. We let *n*=100,200, or 400. We let *ρ*=0.5, which corresponds to a weak correlation between responses as the average absolute correlation between responses is only 0.36. Another value of *ρ* is 0.9, which corresponds to a moderate correlation between responses as the average absolute correlation between responses is about 0.50.

In total, we have six model conditions, and for each model condition, we generate 200 datasets. To evaluate the prediction accuracy of the various methods, we draw 200 additional subjects as testing data. The true correlation functions and a sample of the simulated data are shown in the Supporting Information.

We compare mFACEs and mFPCA in terms of estimation accuracy of the covariance functions, the eigenfunctions and eigenvalues, and prediction of new subjects. For covariance function estimation, we use the relative integrated square errors (RISE). Let ${Ĉ}_{kk^{\prime }}(s,t)$ be an estimate of $C_{kk^{\prime }}(s,t)$, and then RISE are given by

$$\frac{\sum_{k=1}^{p}\sum_{k^{\prime }=1}^{p}\int_{0}^{1}\int_{0}^{1}{\left\{C_{kk^{\prime }}(s,t)-{Ĉ}_{kk^{\prime }}(s,t)\right\}}^{2}\;dsdt}{\sum_{k=1}^{p}\sum_{k^{\prime }=1}^{p}\int_{0}^{1}\int_{0}^{1}{\left\{C_{kk^{\prime }}(s,t)\right\}}^{2}\;dsdt}.$$

For estimating the *ℓ*th eigenfunction, we use the integrated square errors (ISE), which are defined as

$$\min\left[\overset{p}{\underset{k=1}{\sum }}\int_{0}^{1}{\left\{\Psi_{\ell }^{\left(k\right)}(t)-{\hat{\Psi }}_{\ell }^{\left(k\right)}(t)\right\}}^{2}\;dt,\;\;\overset{p}{\underset{k=1}{\sum }}\int_{0}^{1}{\left\{\Psi_{\ell }^{\left(k\right)}(t)+{\hat{\Psi }}_{\ell }^{\left(k\right)}(t)\right\}}^{2}\;dt\right].$$

Note that the range of ISE is [0,2]. For estimating the eigenvalues, we use the ratio of the estimate against the truth; that is, ${\hat{d}}_{\ell }/d_{\ell }$.

For predicting new curves, we use the mean integrated square errors (MISE), which are given by

$$\frac{1}{200p}\overset{p}{\underset{k=1}{\sum }}\overset{200}{\underset{i=1}{\sum }}\left[\int_{0}^{1}{\left\{x_{i}^{\left(k\right)}(t)-{\hat{x}}_{i}^{\left(k\right)}(t)\right\}}^{2}\;dt\right].$$

For the curve prediction using mFPCA, we truncate the number of principal components using a PVE of 0.99. It is worth noting that if no truncation is adopted, then the curve prediction using mFPCA reduces to curve prediction using univariate FPCA. We shall also consider the conditional expectation method based on the estimates of covariance functions from mFPCA. The method is denoted by mFPCA(CE), and its difference with mFACEs is that different estimates of covariance functions are used.

### 3.2 Simulation results

**Figure 1:**
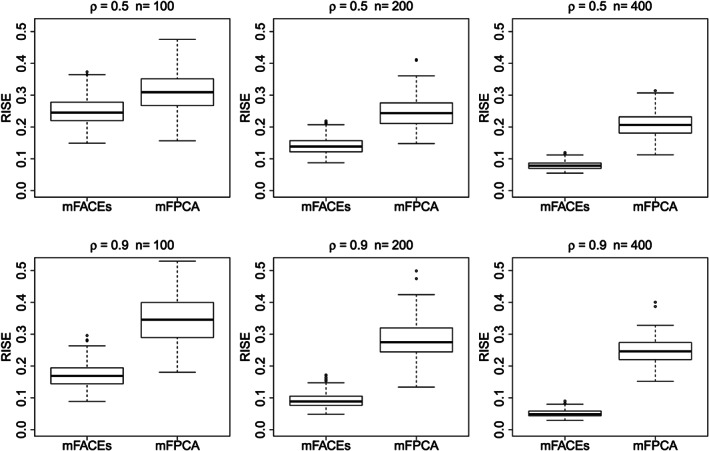
Boxplots of relative integrated square errors (RISEs) of mFACEs and mFPCA for estimating the covariance function

Figure 1 gives boxplots of RISEs of mFACEs and mFPCA for estimating covariance functions. Under all model conditions, mFACES outperforms mFPCA, and the improvement in RISEs as the sample size increases is much more pronounced for mFACEs. Under the model conditions with moderate correlations (*ρ*=0.9), the advantage of mFACEs is substantial even for the small sample size *n*=100.

**Figure 2:**
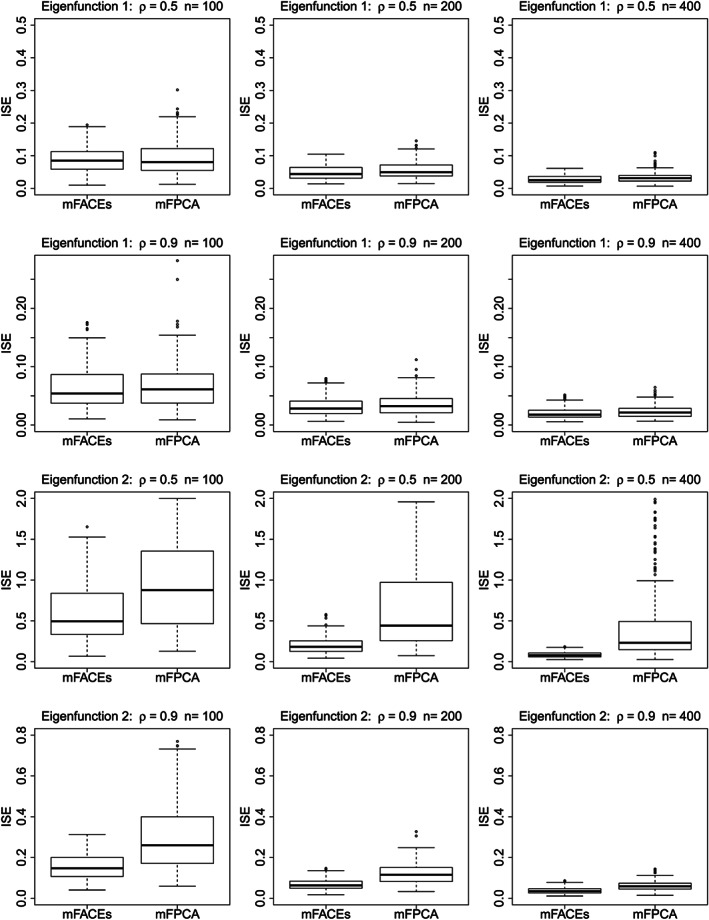
Boxplots of integrated square errors (ISEs) of mFACEs and mFPCA for estimating the top two eigenfunctions

**Figure 3:**
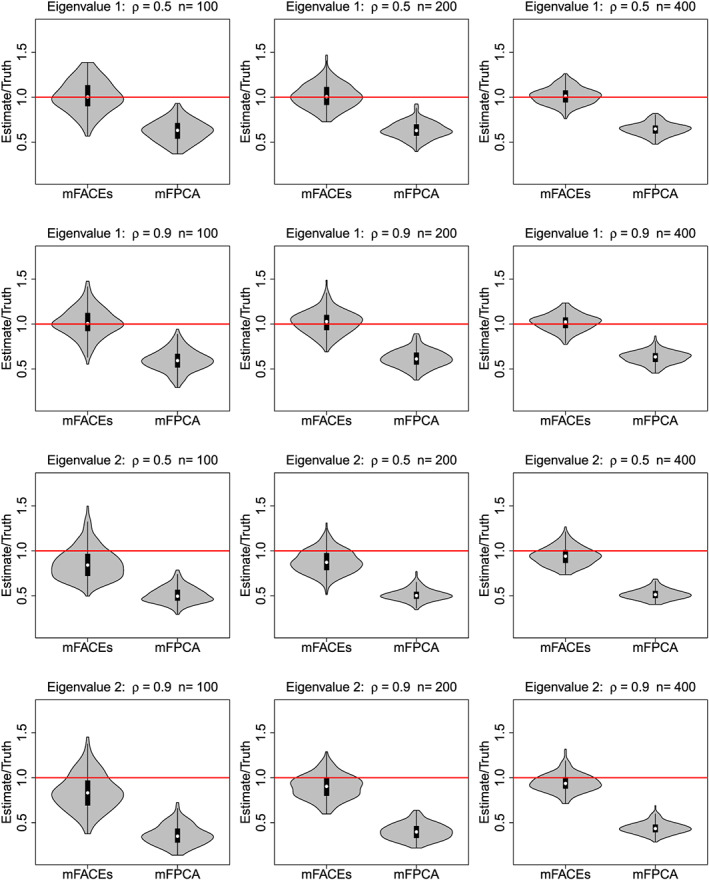
Violin plots of mFACEs and mFPCA for estimating the top two eigenvalues. The red horizontal lines indicate that the estimates are equal to the truth

Figures 2 and 3 give boxplots of ISEs and violin plots of mFACEs and mFPCA for estimating the top two eigenfunctions and eigenvalues, respectively. The top two eigenvalues account for about 60*%* of the total variation in the functional data for *ρ*=0.5, and it is 80*%* for *ρ*=0.9. Figure 2 shows that although the two methods are overall comparable for estimating the first eigenfunction, mFACEs has a much better accuracy for estimating the second eigenfunction than mFPCA. The violin plots in Figure 3 show that mFACEs outperforms mFPCA substantially for estimating both eigenvalues under all model conditions. The mFPCA always underestimates the eigenvalues as the variation of scores from univariate FPCA is smaller than the true variation and hence leads to underestimates of eigenvalues.

**Figure 4:**
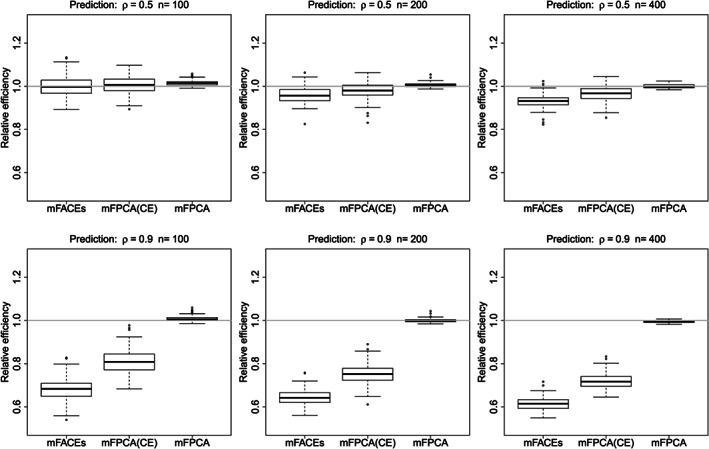
Boxplots of relative efficiency of three methods for curve prediction. The grey horizontal lines indicate the mean integrated square errors (MISEs) for univariate functional principal component analysis (FPCA)

Finally, we consider the prediction of new subjects by mFACEs, mFPCA, and mFPCA(CE). We define the relative efficiencies of different methods as the ratios of MISEs with respect to those of univariate FPCA; see Figure 4. Univariate FPCA is implemented in the R package face (Xiao, Li, Checkley, & Crainiceanu, 2018). We have the following findings. Under all model conditions, mFACEs has the smallest MISE, mFPCA(CE) has the second best performance, and mFPCA is close to univariate FPCA. Thus, on average, mFACEs provides the most accurate curve prediction. These results indicate that (a) mFACEs has better covariance estimation than mFPCA(CE) and so is the prediction based on it; (b) compared with mFPCA/univariate FPCA, mFPCA(CE) exploits the correlation information and hence results in better predictions.

In summary, mFACEs shows competing performance against alternative methods.

## 4 APPLICATION TO ALZHEIMER'S DISEASE STUDY

The ADNI is a two‐stage longitudinal observational study launched in year 2003 with the primary goal of investigating whether serial neuroimages, biological markers, clinical and neuropsychological assessments can be combined to measure the progression of AD (Weiner et al., 2017). The ADNI‐1 data from the first stage contain 379 patients with amnestic mild cognitive impairment (MCI, a risk state for AD) at baseline who had at least one follow‐up visit. Participants were assessed at baseline, 6, 12, 18, 24, and 36 months with additional annual follow‐ups included in the second stage of the study. At each visit, various neuropsychological assessments, clinical measures, and brain images were collected. The ADNI‐2 data include 424 additional patients suffering from MCI and significant memory concern, with at least one follow‐up visit and longitudinal data collected over 4 years. Thus, for the combined data, the total number of subjects is 803, and the average number of visits is 4.72. The data are publicly available at http://ida.loni.ucla.edu/.

We consider five longitudinal markers commonly measured in studies of AD with strong comparative predictive value (Li, Chan, Doody, Quinn, & Luo, 2017). Among the five markers, Disease Assessment Scale‐Cognitive 13 items (ADAS‐Cog 13), Rey Auditory Verbal Learning Test immediate recall (RAVLT.imme), Rey Auditory Verbal Learning Test learning curve (RAVLT.learn), and Mini‐Mental State Examination (MMSE) are neuropsychological assessments. Functional Assessment Questionnaire (FAQ) is a functional and behavioural assessment. High values of ADAS‐Cog 13 and FAQ indicate a high‐risk state for AD, whereas low values of RAVLT.imme, RAVLT.learn, and MMSE reflect severe cognitive impairment. The longitudinal trajectories in ADNI‐1 and ADNI‐2 are defined on the same time domain with the largest follow‐up time of 96 months from the start of ADNI‐1 (Time 0).

### 4.1 Multivariate FPCA via mFACEs

**Figure 5:**
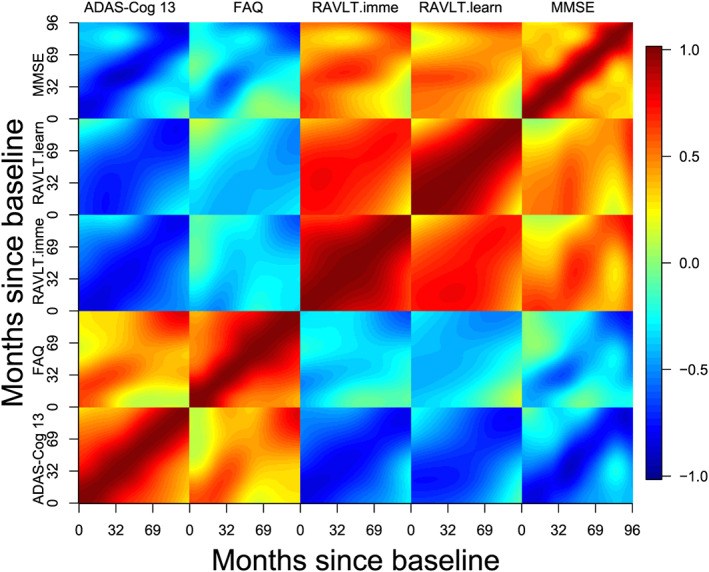
Estimated correlation functions for the longitudinal markers. ADAS‐Cog 13, Disease Assessment Scale‐Cognitive 13 items; FAQ, Functional Assessment Questionnaire; MMSE, Mini‐Mental State Examination; RAVLT.imme, Rey Auditory Verbal Learning Test immediate recall; RAVLT.learn, Rey Auditory Verbal Learning Test learning curve

**Figure 6:**
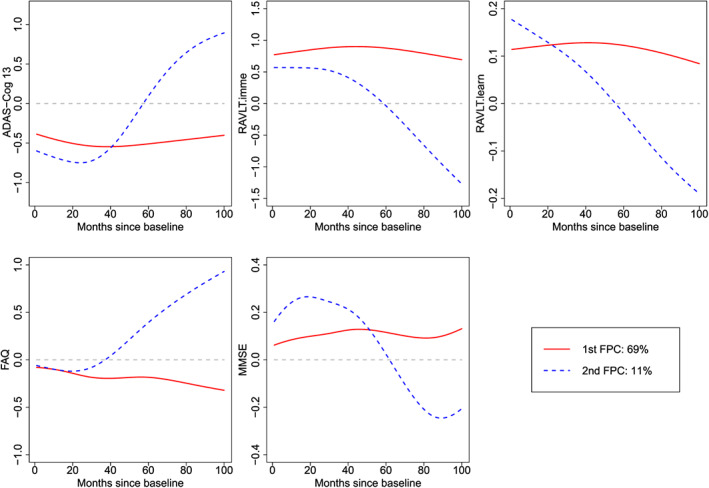
Estimated top two eigenfunctions for the longitudinal markers. ADAS‐Cog 13, Disease Assessment Scale‐Cognitive 13 items; FAQ, Functional Assessment Questionnaire; MMSE, Mini‐Mental State Examination; RAVLT.imme, Rey Auditory Verbal Learning Test immediate recall; RAVLT.learn, Rey Auditory Verbal Learning Test learning curve

**Figure 7:**
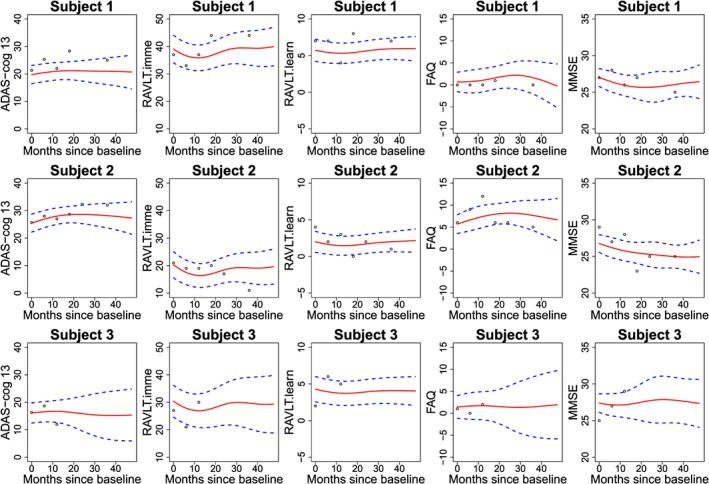
Predicted subject‐specific curves (red solid line) of the longitudinal markers and associated 95*%* point‐wise confidence bands (blue dashed line) for three subjects. ADAS‐Cog 13, Disease Assessment Scale‐Cognitive 13 items; FAQ, Functional Assessment Questionnaire; MMSE, Mini‐Mental State Examination; RAVLT.imme, Rey Auditory Verbal Learning Test immediate recall; RAVLT.learn, Rey Auditory Verbal Learning Test learning curve

We analyse the five longitudinal biomarkers using mFACEs. For better visualization, we plot in Figure 5 the estimated correlation functions $\rho_{kk^{\prime }}(s,t)=C_{kk^{\prime }}(s,t)/\sqrt{C_{kk}(s,s)C_{k^{\prime }k^{\prime }}(t,t)}$. The plot indicates two groups of biomarkers, ADAS‐Cog 13 and FAQ, in one group and RAVLT.imme, RAVLT.learn, and MMSE in another group. The biomarkers within the groups are positively correlated and negatively correlated between groups, which makes sense as high values of ADAS‐Cog 13 and FAQ and low values for the other biomarkers suggest of AD. Next, we display in Figure 6 the two estimated (multivariate) eigenfunctions associated with the top two estimated eigenvalues, which account for 69*%* and 11*%* of the total variance in the functional part of the data. The eigenfunctions reveal how the five biomarkers covary and how a subject's trajectories of biomarkers deviate from the population mean. Indeed, we see from Figure 6 that the first eigenfunction (solid curves) is below the zero line for ADAS‐Cog 13 and FAQ and above the zero line for the other three biomarkers. This means that the score corresponding to the first eigenfunction might be used as an indicator of AD. Indeed, a negative score for the first eigenfunction means higher‐than‐population‐mean values of the former whereas lower‐than‐population‐mean values of the latter, indicating more severe AD status. The second eigenfunction (dashed curves) for the five biomarkers is below the zero line at first and then above it or the other way around, potentially suggesting of a longitudinal pattern of the AD progression. Specifically, these subjects with a positive score for the second eigenfunction will have higher ADAS‐Cog 13/FAQ and lower RAVLT and MMSE over the months, suggesting of AD progression. Finally, we illustrate in Figure 7 the predicted curves along with the associated 95*%* point‐wise confidence bands for three subjects. We focus on predicting the trajectories over the first 4 years as there are more observations. We can see that the confidence bands are getting wider at the later time points because of fewer observations.

### 4.2 Comparison of prediction performance of different methods

**Figure 8:**
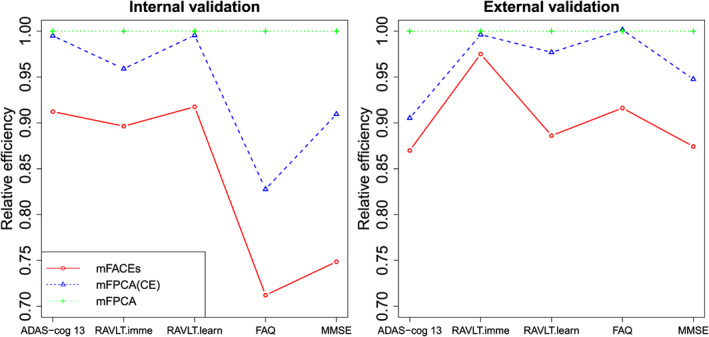
The internal and external prediction validations for the Alzheimer's Disease Neuroimaging Initiative longitudinal makers. ADAS‐Cog 13, Disease Assessment Scale‐Cognitive 13 items; FAQ, Functional Assessment Questionnaire; MMSE, Mini‐Mental State Examination; RAVLT.imme, Rey Auditory Verbal Learning Test immediate recall; RAVLT.learn, Rey Auditory Verbal Learning Test learning curve

We compare the proposed mFACEs with mFPCA and mFPCA(CE) for predicting the five longitudinal biomarkers. The prediction performance is evaluated by the average squared prediction errors (APE),

$${\text{APE}}_{k}=\frac{1}{n}\overset{n}{\underset{i=1}{\sum }}\left[\frac{1}{m_{ik}}\overset{m_{ik}}{\underset{j=1}{\sum }}{\left\{y_{ij}^{\left(k\right)}-{ŷ}_{ij}^{\left(k\right)}\right\}}^{2}\right],$$

where ${ŷ}_{ij}^{\left(k\right)}$ is the predicted value of the *k*th biomarker for the *i*th subject at time $t_{ij}^{\left(k\right)}$. We conduct two types of validation: an internal validation and an external validation. For the internal validation, we perform a 10‐fold cross‐validation to the combined data of ADNI‐1 and ADNI‐2. For the external validation, we fit the model using only the ADNI‐1 data and then predict ADNI‐2 data. Figure 8 summarizes the results. For simplicity, we present the relative efficiency of APE, which is the ratio of APEs of one method against the mFPCA. In both cases, mFACEs achieves better prediction accuracy than competing methods. Note that mFPCA(CE) outperforms mFPCA for predicting almost all biomarkers. The results suggest that (a) mFACEs is better than competing methods for analysing the longitudinal biomarkers. and (b) exploiting the correlations between the biomarkers improves prediction.

## 5 DISCUSSION

The prevalence of multivariate functional data has sparked much research interests in recent years. However, covariance estimation for multivariate sparse functional data remains underdeveloped. We proposed a new method, mFACEs, and its features include the following: (a) A covariance smoothing framework is proposed to tackle multivariate sparse functional data; (b) an automatic and fast fitting algorithm is adopted to ensure the scalability of the method; (c) eigenfunctions and eigenvalues can be obtained through a one‐time spectral decomposition, and eigenfunctions can be easily evaluated at any sampling points; and (d) a multivariate extension of the conditional expectation approach (Yao et al., 2005) is derived to exploit correlations between outcomes. The simulation study and the data example showed that mFACEs could better capture between‐function correlations and thus gives improved principal component analysis and curve prediction.

When the magnitude of functional data are quite different, one may first normalize the functional data, as recommended by Chiou et al. (2014). One method of normalization is to rescale the functional data using the estimated variance function ${Ĉ}_{kk}{\left(t,t\right)}^{-1/2}$ as in Chiou et al. (2014) and Jacques and Preda (2014). An alternative method is to use a global rescaling factor like ${\left(\int {Ĉ}_{kk}(t,t)\;dt\right)}^{-1/2}$ as in Happ and Greven (2018). Both methods can be easily incorporated into our proposed method. In our data analysis, we find that the results with normalization are very close to those without normalization; thus, we present the results without normalization.

Because multivariate FPCA is more complex than univariate FPCA, weak correlations between the functions and small sample size may offset the benefit of conducting multivariate FPCA; see Section 7.3 in Wong et al. (2019). Thus, it is of future interest to develop practical tests to determine if correlations between multivariate functional data are different from 0.

The mFACEs method has been implemented in an R package mfaces and will be submitted to CRAN for public access.

## DATA AVAILABILITY STATEMENT

Data used in preparation of this article were obtained from the Alzheimer's Disease Neuroimaging Initiative (ADNI) database (http://adni.loni.usc.edu). As such, the investigators within the ADNI contributed to the design and implementation of ADNI and/or provided data but did not participate in analysis or writing of this paper. A complete listing of ADNI investigators can be found at: http://adni.loni.usc.edu/wp‐content/uploads/how_to_apply/ADNI_Acknowledgement_List.pdf.

## Supporting information



The Supporting Information contains additional details and results of a simulation that mimics the ADNI data. R package mfaces (https://github.com/cli9/mfaces) contains an open‐source implementation of the proposed method described in the article. Demo zip file contains codes to demonstrate the proposed method with a simulated dataset in Section 3.

Supporting info item

## APPENDIX A PROOFS OF PROPOSITIONS 1 AND 2

Define $\tilde{b}(t)=G^{-\frac{1}{2}}b(t)$, and then $\int \tilde{b}(t)\tilde{b}{\left(t\right)}^{⊤}\;dt=I$. Define ${\overset{ˇ}{\Theta }}_{kk^{\prime }}(t)=G^{\frac{1}{2}}\Theta_{kk^{\prime }}G^{\frac{1}{2}}$. According to Equation (1),

$$\begin{array}{ccc} d_{\ell }\Psi_{\ell }^{\left(k\right)}(s) & =\overset{p}{\underset{k^{\prime }=1}{\sum }}\int b{\left(s\right)}^{⊤}\Theta_{kk^{\prime }}b(t)\Psi_{\ell }^{\left(k^{\prime }\right)}(t)\;dt & \\ & =\tilde{b}{\left(s\right)}^{⊤}\overset{p}{\underset{k^{\prime }=1}{\sum }}{\overset{ˇ}{\Theta }}_{kk^{\prime }}\int \tilde{b}(t)\Psi_{\ell }^{\left(k^{\prime }\right)}(t)\;dt. \end{array}$$

Thus, $\Psi_{\ell }^{\left(k\right)}(s)=\tilde{b}{\left(s\right)}^{⊤}u_{\ell }^{\left(k\right)}$ with $u_{\ell }^{\left(k\right)}=d_{\ell }^{-1}\left\{\sum_{k^{\prime }=1}^{p}{\overset{ˇ}{\Theta }}_{kk^{\prime }}\int \tilde{b}(t)\Psi_{\ell }^{\left(k^{\prime }\right)}(t)\;dt\right\}\in R^{c}$ and $u_{\ell }={\left(u_{\ell }^{(1),⊤},\text{…},u_{\ell }^{(p),⊤}\right)}^{⊤}\in R^{pc}$. As $\sum_{k=1}^{p}\int \Psi_{\ell }^{\left(k\right)}(t)\Psi_{\ell^{\prime }}^{\left(k\right)}(t)\;dt=1_{\left\{\ell =\ell^{\prime }\right\}}$, we derive that

(B1)
$$u_{\ell }^{⊤}u_{\ell^{\prime }}=\overset{p}{\underset{k=1}{\sum }}u_{\ell }^{(k),⊤}u_{\ell^{\prime }}^{\left(k\right)}=1_{\left\{\ell =\ell^{\prime }\right\}}.$$

By Equation (2),

$$C_{kk^{\prime }}(s,t)=\tilde{b}{\left(s\right)}^{⊤}{\overset{ˇ}{\Theta }}_{kk^{\prime }}\tilde{b}(t)=\tilde{b}{\left(s\right)}^{⊤}\left\{\underset{\ell \geq 1}{\sum }d_{\ell }u_{\ell }^{\left(k\right)}u_{\ell }^{(k^{\prime }),⊤}\right\}\tilde{b}(t),$$

which gives

$${\overset{ˇ}{\Theta }}_{kk^{\prime }}=\underset{\ell \geq 1}{\sum }d_{\ell }u_{\ell }^{\left(k\right)}u_{\ell }^{\left(k^{\prime }\right)}.$$

As 1 ≤ *k*,*k* *′* ≤ *p*, the above is equivalent to

$$\begin{array}{cc} \underset{:=\overset{ˇ}{\Theta }}{\underbrace{\left(\begin{array}{ccc} {\overset{ˇ}{\Theta }}_{11} & \text{…} & {\overset{ˇ}{\Theta }}_{1p}\\ \vdots & \ddots & \vdots \\ {\overset{ˇ}{\Theta }}_{p1} & \text{…} & {\overset{ˇ}{\Theta }}_{pp} \end{array}\right)}} & =\underset{\ell \geq 1}{\sum }d_{\ell }u_{\ell }u_{\ell }^{⊤}. \end{array}$$

Because of Equation (B1), **u**_*ℓ*_s are orthonormal eigenvectors of $\overset{ˇ}{\Theta }$ with *d*_*ℓ*_s the corresponding eigenvalues. The proof is now complete.

By Equation (10),

(B2)
$$\text{iGCV}={\left\|\hat{C}\right\|}^{2}-2f^{⊤}\sum^{-1}f+f^{⊤}\sum^{-2}f+2\overset{n}{\underset{i=1}{\sum }}{\left(L_{i}\sum^{-1}f-f_{i}\right)}^{⊤}\sum^{-1}\left(L_{i}\sum^{-1}f-f_{i}\right).$$

Since $\sum^{-1}=U\text{diag}(\tilde{d})U^{⊤}$, we have

(B3)
$$f^{⊤}\sum^{-1}f={\tilde{f}}^{⊤}\text{diag}(\tilde{d})\tilde{f}={\tilde{d}}^{⊤}(\tilde{f}⊙\tilde{f}).$$

Similarly,

(B4)
$$f^{⊤}\sum^{-2}f={\tilde{f}}^{⊤}\text{diag}({\tilde{d}}^{2})\tilde{f}={\left(\tilde{f}⊙\tilde{d}\right)}^{⊤}(\tilde{f}⊙\tilde{d}).$$

Next we derive that

$$\begin{array}{c} {\left(L_{i}\sum^{-1}f-f_{i}\right)}^{⊤}\sum^{-1}\left(L_{i}\sum^{-1}f-f_{i}\right)={\left(U^{⊤}L_{i}\sum^{-1}f-U^{⊤}f_{i}\right)}^{⊤}\text{diag}(\tilde{d})\left(U^{⊤}L_{i}\sum^{-1}f-U^{⊤}f_{i}\right)={\left({\tilde{L}}_{i}\text{diag}(\tilde{d})\tilde{f}-{\tilde{f}}_{i}\right)}^{⊤}\text{diag}(\tilde{d})\left({\tilde{L}}_{i}\text{diag}(\tilde{d})\tilde{f}-{\tilde{f}}_{i}\right). \end{array}$$

It follows that

(B5)
$$\begin{array}{c} {\left(L_{i}\sum^{-1}f-f_{i}\right)}^{⊤}\sum^{-1}\left(L_{i}\sum^{-1}f-f_{i}\right)={\tilde{d}}^{⊤}\left[\left\{{\tilde{L}}_{i}(\tilde{f}⊙\tilde{d})\right\}⊙\left\{{\tilde{L}}_{i}(\tilde{f}⊙\tilde{d})\right\}+({\tilde{f}}_{i}⊙{\tilde{f}}_{i})\right]-2{\tilde{d}}^{⊤}\left\{({\tilde{f}}_{i}{\tilde{f}}^{⊤})⊙{\tilde{L}}_{i}\right\}\tilde{d}. \end{array}$$

By combining Equations  (B2), (B3), (B4), and (B5), the proof is complete.

## APPENDIX B LEMMA

Lemma 1 The covariance operator with the covariance functions defined in Section 4.1 is positive semidefinite.

Let $a={\left(a_{1},\text{…},a_{p}\right)}^{⊤}\in R^{p}$ and $\tilde{X}=a^{⊤}x$, and then $\tilde{X}$ is a stochastic process with covariance function

$$\text{Cov}\left\{\tilde{X}(s),\tilde{X}(t)\right\}=\underset{kk^{\prime }}{\sum }a_{k}a_{k^{\prime }}C_{kk^{\prime }}(s,t)=\underset{kk^{\prime }}{\sum }a_{k}a_{k^{\prime }}\left(\rho +(1-\rho )1_{\left\{k=k^{\prime }\right\}}\right){\tilde{\Phi }}_{k}{\left(s\right)}^{⊤}{\tilde{\Phi }}_{k^{\prime }}(t),$$

where ${\tilde{\Phi }}_{k}(s)=\Phi_{k}{\left(s\right)}^{⊤}\Lambda_{kk}^{\frac{1}{2}}$. Let $\Psi (s)=\sum_{k=1}^{p}a_{k}{\tilde{\Phi }}_{k}(s)$. Then

$$\text{Cov}\left\{\tilde{X}(s),\tilde{X}(t)\right\}=\rho \Psi {\left(s\right)}^{⊤}\Psi (t)+(1-\rho )\underset{k}{\sum }a_{k}^{2}{\tilde{\Phi }}_{k}{\left(s\right)}^{⊤}{\tilde{\Phi }}_{k}(t),$$

which is always positive semidefinite, and the proof is complete.

## References

[sta4245-bib-0001] Berrendero, J. , Justel, A. , & Svarc, M. (2011). Principal components for multivariate functional data. Computational Statistics Data Analysis, 55(9), 2619–2634.

[sta4245-bib-0002] Cai, T. , & Yuan, M. (2010). Nonparametric covariance function estimation for functional and longitudinal data. University of Pennsylvania and Georgia Institute of Technology.

[sta4245-bib-0003] Chiou, J. M. , Chen, Y. T. , & Yang, Y. F. (2014). Multivariate functional principal component analysis: A normalization approach. Statistica Sinica, 24(4), 1571–1596.

[sta4245-bib-0004] Chiou, J. M. , & Müller, H. G. (2014). Linear manifold modelling of multivariate functional data. Journal of the Royal Statistical Society: Series B (Statistical Methodology), 76(3), 605–626.

[sta4245-bib-0005] Chiou, J. M. , & Müller, H. G. (2016). A pairwise interaction model for multivariate functional and longitudinal data. Biometrika, 103(2), 377–396. 27279664 10.1093/biomet/asw007

[sta4245-bib-0006] Eilers, P. , & Marx, B. (1996). Flexible smoothing with B‐splines and penalties (with discussion). Statistical Science, 11(2), 89–121.

[sta4245-bib-0007] Eilers, P. , & Marx, B. (2003). Multivariate calibration with temperature interaction using two‐dimensional penalized signal regression. Chemometrics and Intelligent Laboratory Systems, 66, 159–174.

[sta4245-bib-0008] Goldsmith, J. , Crainiceanu, C. M. , Caffo, B. , & Reich, D. (2012). Longitudinal penalized functional regression for cognitive outcomes on neuronal tract measurements. Journal of the Royal Statistical Society: Series C (Applied Statistics), 61(3), 453–469. 22679339 10.1111/j.1467-9876.2011.01031.x PMC3366511

[sta4245-bib-0009] Greven, S. , Crainiceanu, C. M. , Caffo, B. , & Reich, D. (2010). Longitudinal functional principal component. Electronic Journal of Statistical, 4, 1022–1054. 10.1214/10-EJS575 PMC3131008 21743825

[sta4245-bib-0010] Happ, C. , & Greven, S. (2018). Multivariate functional principal component analysis for data observed on different (dimensional) domains. Journal of the American Statistical Association, 113(522), 649–659.

[sta4245-bib-0011] Huang, H. , Li, Y. , & Guan, Y. (2014). Joint modeling and clustering paired generalized longitudinal trajectories with application to cocaine abuse treatment data. Journal of the American Statistical Association, 109(508), 1412–1424.

[sta4245-bib-0012] Jacques, J. , & Preda, C. (2014). Model‐based clustering for multivariate functional data. Computational Statistics & Data Analysis, 71, 92–106.

[sta4245-bib-0013] James, G. , Hastie, T. , & Sugar, C. (2000). Principal component models for sparse functional data. Biometrika, 87(3), 587–602.

[sta4245-bib-0014] Kowal, D. R. , Matteson, D. S. , & Ruppert, D. (2017). A Bayesian multivariate functional dynamic linear model. Journal of the American Statistical Association, 112(518), 733–744.

[sta4245-bib-0015] Leng, X. , & Müller, H. (2006). Classification using functional data analysis for temporal gene expression data. Bioinformatics, 22(1), 68–76. 16257986 10.1093/bioinformatics/bti742

[sta4245-bib-0016] Li, K. , Chan, W. , Doody, R. S. , Quinn, J. , & Luo, S. (2017). Prediction of conversion to Alzheimer's disease with longitudinal measures and time‐to‐event data. Journal of Alzheimer's Disease, 58(2), 361–371. 10.3233/JAD-161201 PMC5477671 28436391

[sta4245-bib-0017] Li, J. , Huang, C. , & Zhu, H. (2017). A functional varying‐coefficient single‐index model for functional response data. Journal of the American Statistical Association, 112(519), 1169–1181. 29200540 10.1080/01621459.2016.1195742 PMC5710774

[sta4245-bib-0018] Li, Y. , Wang, N. , & Carroll, R. J. (2013). Selecting the number of principal components in functional data. Journal of the American Statistical Association, 108(504), 1284–1294. 10.1080/01621459.2013.788980 PMC3872138 24376287

[sta4245-bib-0019] Lindquist, M. (2012). Functional causal mediation analysis with an application to brain connectivity. Journal of the American Statistical Association, 107(500), 1297–1309. 25076802 10.1080/01621459.2012.695640 PMC4112546

[sta4245-bib-0020] Luo, R. , & Qi, X. (2017). Function‐on‐function linear regression by signal compression. Journal of the American Statistical Association, 112(518), 690–705.

[sta4245-bib-0021] Morris, J. , Arroyo, C. , Coull, B. , Ryan, L. , Herrick, R. , & Gortmaker, S. (2006). Using wavelet‐based functional mixed models to characterize population heterogeneity in accelerometer profiles: A case study. Journal of the American Statistical Association, 101(476), 1352–1364. 19169424 10.1198/016214506000000465 PMC2630189

[sta4245-bib-0022] Park, J. , & Ahn, J. (2017). Clustering multivariate functional data with phase variation. Biometrics, 73(1), 324–333. 27218696 10.1111/biom.12546

[sta4245-bib-0023] Peng, J. , & Paul, D. (2009). A geometric approach to maximum likelihood estimation of functional principal components from sparse longitudinal data. Journal of Computational and Graphical Statistics, 18(4), 995–1015.

[sta4245-bib-0024] Petersen, A. , & Müller, H. G. (2016). Fréchet integration and adaptive metric selection for interpretable covariances of multivariate functional data. Biometrika, 103(1), 103–120.

[sta4245-bib-0025] Qi, X. , & Luo, R. (2018). Function‐on‐function regression with thousands of predictive curves. Journal of Multivariate Analysis, 163, 51–66.

[sta4245-bib-0026] Qiao, X. , Guo, S. , & James, G. M. (2019). Functional graphical models. Journal of the American Statistical Association, 114(525), 211–222.

[sta4245-bib-0027] Ramsay, J. , & Silverman, B. (2005). Functional data analysis. New York: Springer.

[sta4245-bib-0028] Reimherr, M. , & Nicolae, D. (2014). A functional data analysis approach for genetic association studies. The Annals of Applied Statistics, 8(1), 406–429.

[sta4245-bib-0029] Reimherr, M. , & Nicolae, D. (2016). Estimating variance components in functional linear models with applications to genetic heritability. Journal of the American Statistical Association, 111(513), 407–422.

[sta4245-bib-0030] Reiss, P. , & Ogden, R. (2010). Functional generalized linear models with images as predictors. Biometrics, 66(1), 61–69. 19432766 10.1111/j.1541-0420.2009.01233.x

[sta4245-bib-0031] Saporta, G. (1981). Méthodes exploratoires d'analyse de données temporelles. Cahiers du Bureau universitaire de recherche opérationnelle Série Recherche, 37, 7–194.

[sta4245-bib-0032] Seber, G. (2008). A matrix handbook for statisticians. Hoboken, New Jersey: John Wiley & Sons.

[sta4245-bib-0033] Weiner, M. W. , Veitch, D. P. , Aisen, P. S. , Beckett, L. A. , Cairns, N. J. , Green, R. C. , & Petersen, R. C. (2017). Recent publications from the Alzheimer's disease neuroimaging initiative: Reviewing progress toward improved ad clinical trials. Alzheimer's & Dementia: The Journal of the Alzheimer's Association, 13(4), e1–e85. 10.1016/j.jalz.2016.11.007 PMC6818723 28342697

[sta4245-bib-0034] Wong, R. K. , Li, Y. , & Zhu, Z. (2019). Partially linear functional additive models for multivariate functional data. Journal of the American Statistical Association, 114(525), 406–418.

[sta4245-bib-0035] Wong, R. K. , & Zhang, X. (2019). Nonparametric operator‐regularized covariance function estimation for functional data. Computational Statistics & Data Analysis, 131, 131–144.

[sta4245-bib-0036] Wood, S. N. (2000). Modelling and smoothing parameter estimation with multiple quadratic penalties. Journal of the Royal Statistical Society: Series B (Statistical Methodology), 62(2), 413–428.

[sta4245-bib-0037] Xiao, L. , Huang, L. , Schrack, J. , Ferrucci, L. , Zipunnikov, V. , & Crainiceanu, C. M. (2015). Quantifying the life‐time circadian rhythm of physical activity: A covariate‐dependent functional approach. Biostatistics, 16(2), 352–367. 25361695 10.1093/biostatistics/kxu045 PMC4804116

[sta4245-bib-0038] Xiao, L. , Li, C. , Checkley, W. , & Crainiceanu, C. M. (2018). R package face: Fast covariance estimation for sparse functional data (version 0.1‐4). http://cran.r‐project.org/web/packages/face/index.html. 10.1007/s11222-017-9744-8 PMC5807553 29449762

[sta4245-bib-0039] Xiao, L. , Li, C. , Checkley, W. , & Crainiceanu, C. M. (2018). Fast covariance estimation for sparse functional data. Statistics and Computing, 28(3), 511–522. 29449762 10.1007/s11222-017-9744-8 PMC5807553

[sta4245-bib-0040] Xu, G. , & Huang, J. (2012). Asymptotic optimality and efficient computation of the leave‐subject‐out cross‐validation. Annals of Statistics, 40(6), 3003–3030.

[sta4245-bib-0041] Yao, F. , Müller, H. , & Wang, J. (2005). Functional data analysis for sparse longitudinal data. Journal of the American Statistical Association, 100(470), 577–590.

[sta4245-bib-0042] Zhou, L. , Huang, J. Z. , & Carroll, R. J. (2008). Joint modelling of paired sparse functional data using principal components. Biometrika, 95(3), 601–619. 19396364 10.1093/biomet/asn035 PMC2672432

[sta4245-bib-0043] Zhou, S. , Shen, X. , & Wolfe, D. A. (1998). Local asymptotics for regression splines and confidence regions. Annals of Statistics, 26(5), 1760–1782.

[sta4245-bib-0044] Zhu, H. , Brown, P. , & Morris, J. (2012). Robust classification of functional and quantitative image data using functional mixed models. Biometrics, 68(4), 1260–1268. 22670567 10.1111/j.1541-0420.2012.01765.x PMC3443537

[sta4245-bib-0045] Zhu, H. , Li, R. , & Kong, L. (2012). Multivariate varying coefficient model for functional responses. Annals of Statistics, 40(5), 2634–2666. 23645942 10.1214/12-AOS1045SUPP PMC3641708

[sta4245-bib-0046] Zhu, H. , Morris, J. S. , Wei, F. , & Cox, D. D. (2017). Multivariate functional response regression, with application to fluorescence spectroscopy in a cervical pre‐cancer study. Computational Statistics & Data Analysis, 111, 88–101. 29051679 10.1016/j.csda.2017.02.004 PMC5642121

[sta4245-bib-0047] Zhu, H. , Strawn, N. , & Dunson, D. B. (2016). Bayesian graphical models for multivariate functional data. Journal of Machine Learning Research, 17(1), 7157–7183.

