# Supplementary material for: Fast covariance estimation for multivariate sparse functional data
Source: Stat (Int Stat Inst). 2020 Jun 17;9(1):e245. doi: 10.1002/sta4.245 (PMC8276768; doi:10.1002/sta4.245)
Supplement: Supplementary file 1 — Supporting info item [file STA4-9-0-s001.zip › Supplement.pdf]

Supplement for "Fast Covariance Estimation for Multivariate Sparse Functional Data"
by

Cai Li, Luo Xiao and Sheng Luo

Section S.1 presents details of mean function estimation. Section S.2 shows the true correlation functions and a sample of the simulated data in Section 3.1. Section S.3 provides the results of an additional simulation which mimics the ADNI data.

S.1 Mean Function Estimation

The smooth mean function $\mu^{(k)}(t)$ is approximated by the B-spline basis functions $f^{(k)}(t) = \sum_{1\leq\gamma\leq c} \alpha_\gamma^{(k)} B_\gamma(t)$, where $\boldsymbol{\alpha}_k = \left\{\alpha_1^{(k)}, \ldots, \alpha_c^{(k)}\right\}^\top \in \mathbb{R}^c$ is a coefficient vector. For simplicity, we use the same set of B-spline bases as in the covariance function estimation. We carry out univariate smoothing for each response using P-splines (Eilers & Marx 1996) and $\boldsymbol{\alpha}_k$ is obtained by minimizing

$$\sum_{i=1}^{n}\sum_{j=1}^{m_{ik}} \left\{ f^{(k)}\left(t_{ij}^{(k)}\right) - y_{ij}^{(k)} \right\}^2 + \tau_k \|\mathbf{D}\boldsymbol{\alpha}_k\|^2, \tag{S.1}$$

where τ_k is a nonnegative smoothing parameter to be selected by leave-one-subject-out cross validation for the kth response. Note that the penalty term is essentially equivalent to the integrated squared second derivative of $f^{(k)}$. Denote the minimizer of (S.1) by $\widehat{\boldsymbol{\alpha}}_k$, then the estimate of the mean function $\mu^{(k)}(t)$ is given by $\widehat{\mu}^{(k)}(t) = \sum_{1\leq\gamma\leq c} \widehat{\alpha}_\gamma^{(k)} B_\gamma(t)$.

S.2 Additional Figures

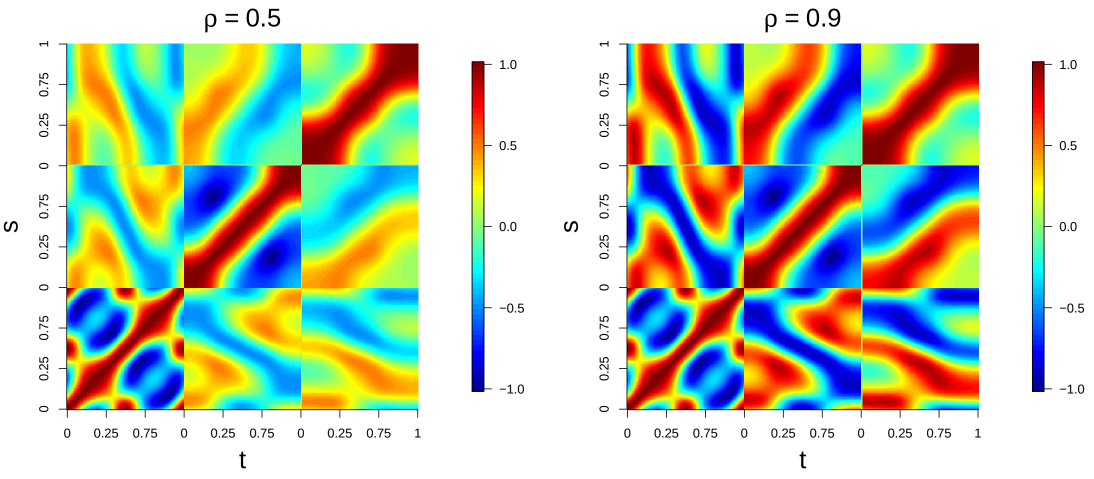

Figure S.1: True correlation functions in Section 3.1.

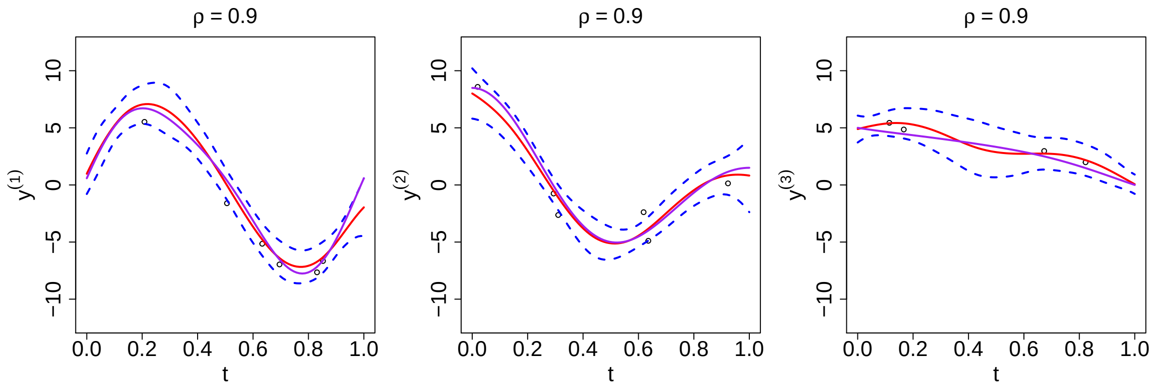

Figure S.2: A sample of the simulated data in Section 3.1. Note that the sampling time points vary between responses. The red solid lines represent the predicted subject-specific curves with $n = 400$, the blue dashed lines are associated 95% point-wise confidence bands, the purple solid lines are the true underlying trajectories.

S.3 Additional Simulation Results

We specify the model according to the estimates of model components through mFACEs. Specifically, there are five responses with the mean functions shown in Fig S.3. The covariance function has 7 principal components, and we use all of them to simulate data. We shall focus on estimating the two leading principal components which account for 80% of the total variation. $\xi_{i\ell}$ are generated from a normal distribution with mean zero and variance d_ℓ for $\ell = 1, \ldots, 7$. The white noise $\epsilon_{ij}^{(k)}$ are sampled from a normal distribution with mean zero and variance σ_k^2 for $k = 1, \ldots, 5$. The corresponding eigenvalues $\{d_\ell\}_{1\leq\ell\leq7}$ are $139.466, 23.121, 18.803, 8.903, 6.801, 1.819, 1.232$, and the error variances $\{\sigma_k^2\}_{1\leq k\leq5}$ are $4.383, 5.945, 1.889, 2.877, 1.658$. In this setting, we assume the same sampling time points between responses within subjects. For each subject i, m_i observations are randomly selected from a set of 100 equidistant sampling points in the unit interval, and the rest of them are masked. We consider four levels of the sample size: $n = 100, 200, 400, 800$. For each sample size, 200 new subjects are drawn as testing data. We generate 200 datasets for each model condition.

References

Eilers, P. & Marx, B. (1996), 'Flexible smoothing with B-splines and penalties (with Discussion)', *Statist. Sci.* **11**, 89–121.

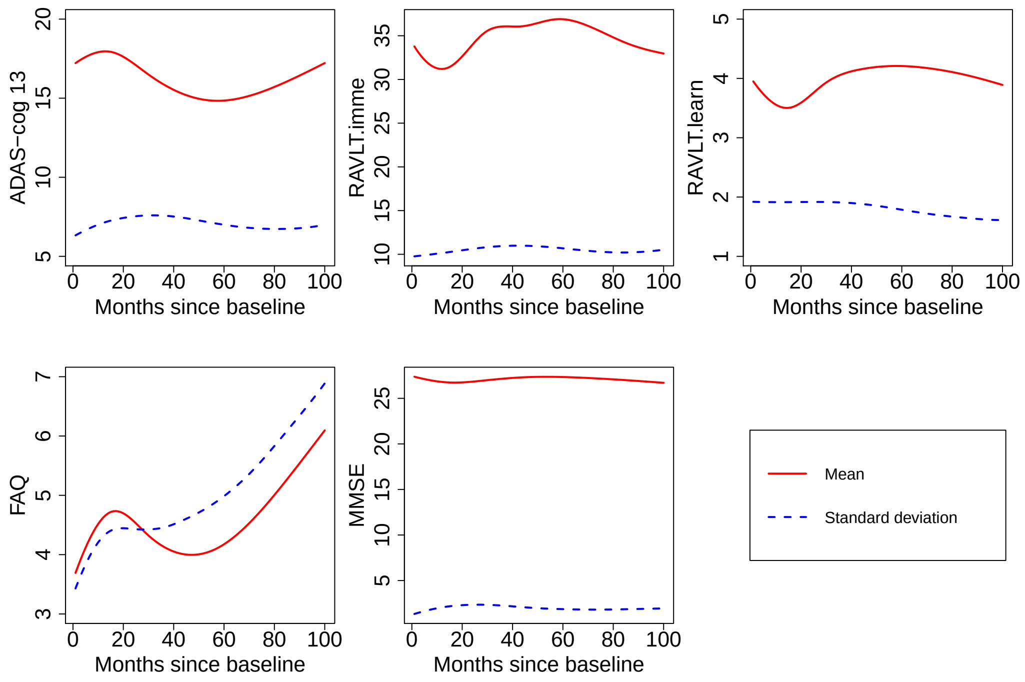

Figure S.3: Estimated mean and standard deviation functions for the longitudinal markers.

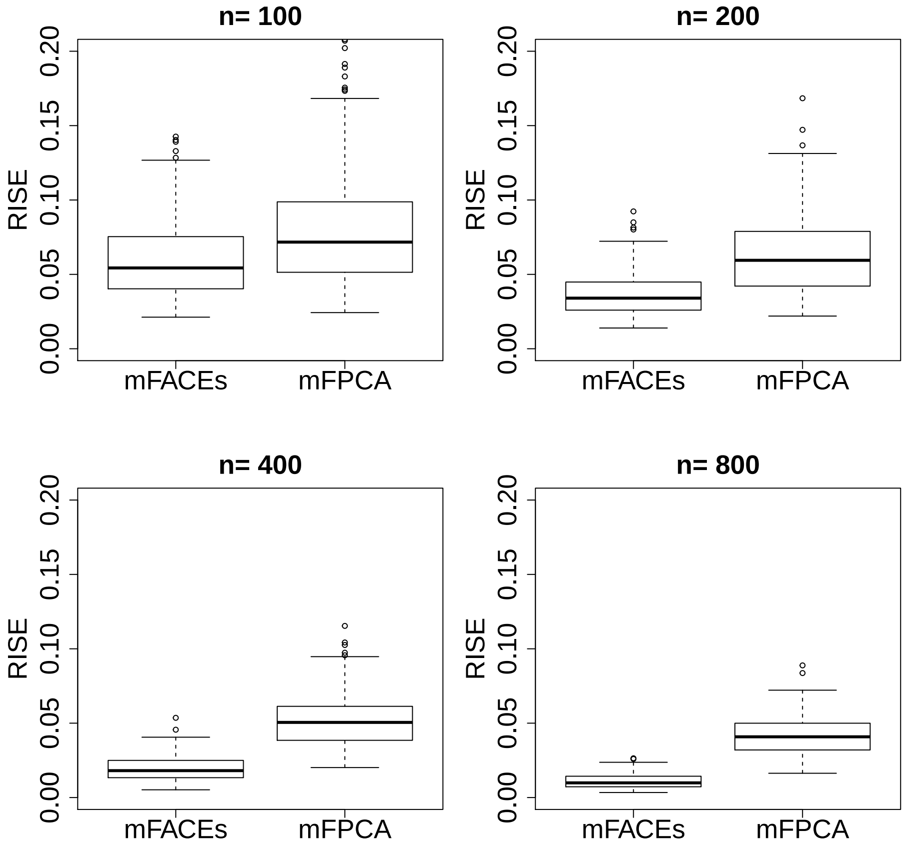

Figure S.4: Boxplots of RISEs of mFACEs and mFPCA for estimating the covariance function.

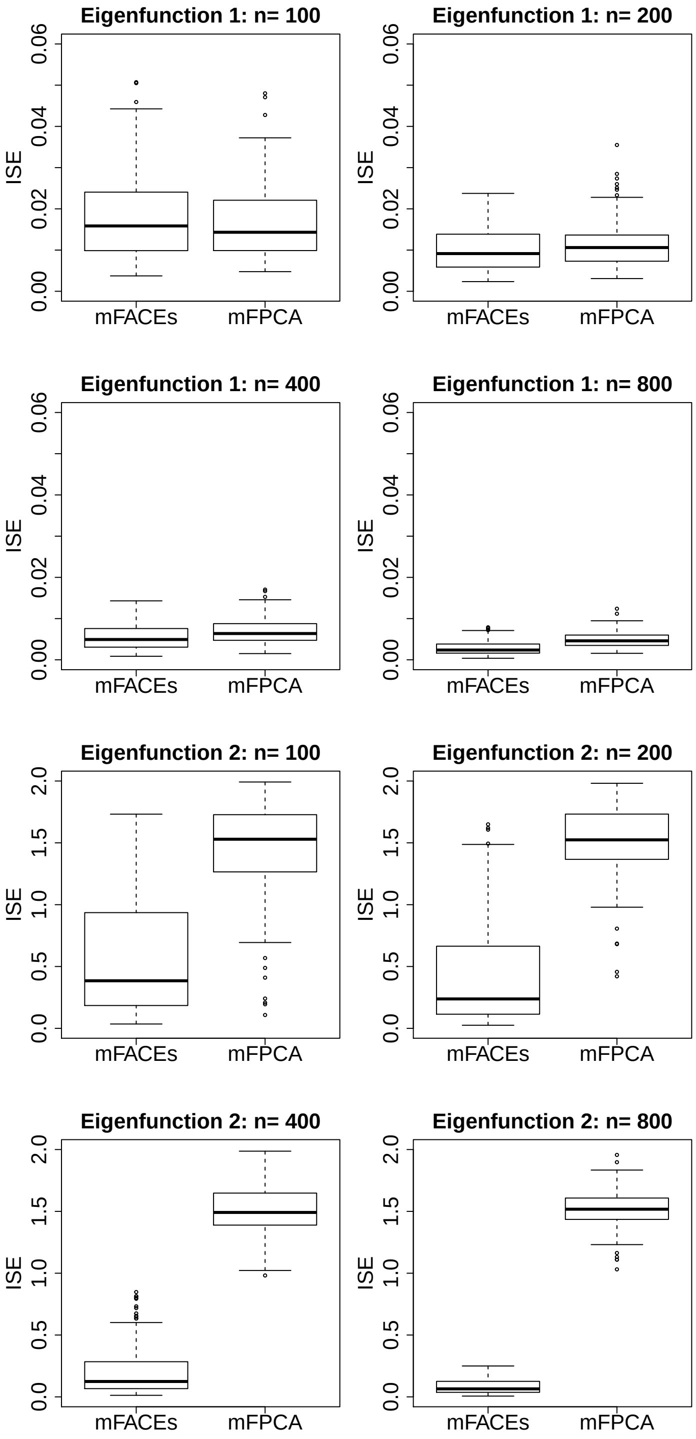

Figure S.5: Boxplots of ISEs of mFACEs and mFPCA for estimating the top two eigenfunctions.

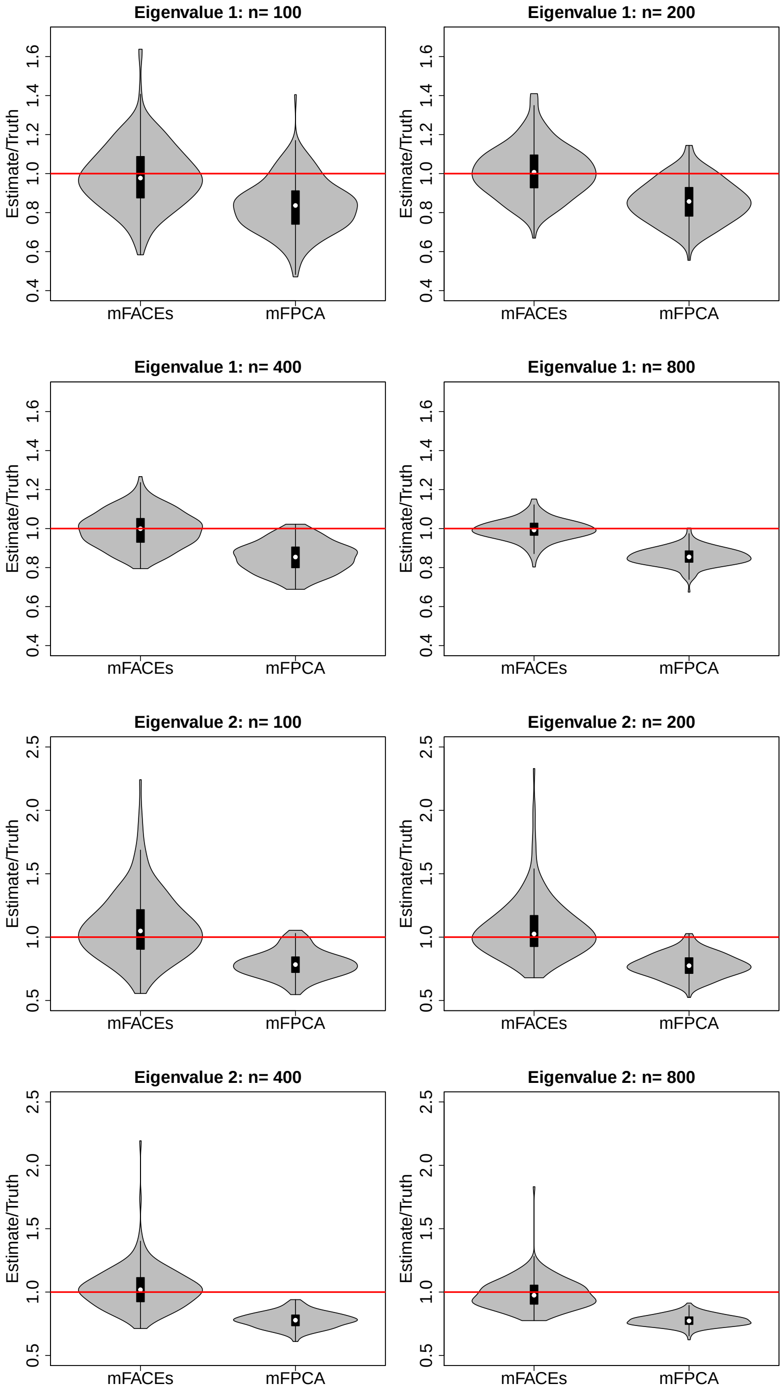

Figure S.6: Violin plots of mFACEs and mFPCA for estimating the top two eigenvalues.

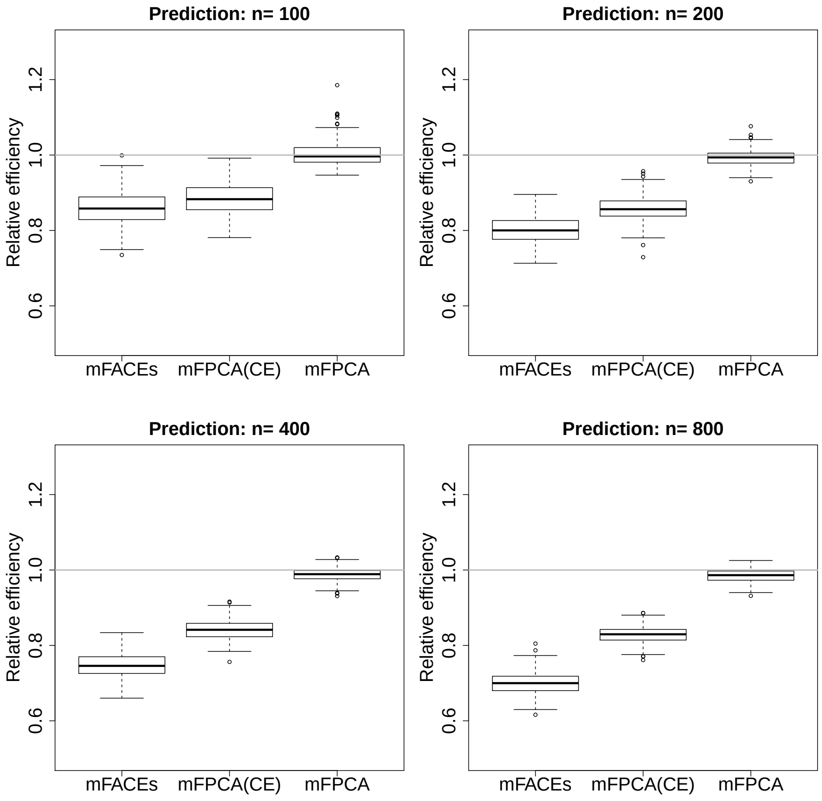

Figure S.7: Boxplots of relative efficiency of three methods for curve prediction.
